# Synthetic pathways to create asymmetric center at C1 position of 1-substituted-tetrahydro-β-carbolines – a review

**DOI:** 10.1039/d4ra05961a

**Published:** 2024-09-19

**Authors:** Md. Moaz Ahmed Asif, Susmita Roy Lisa, Nazmul Qais

**Affiliations:** a Department of Pharmacy, Faculty of Science & Engineering, University of Information Technology & Sciences Holding 190, Road 5, Block J, Baridhara, Maddha Nayanagar, Vatara Dhaka-1212 Bangladesh; b Department of Chemistry, Syracuse University Syracuse NY 13244 USA; c Department of Clinical Pharmacy and Pharmacology, Faculty of Pharmacy, University of Dhaka Dhaka-1000 Bangladesh nqais@du.ac.bd

## Abstract

The 2,3,4,9-tetrahydro-1*H*-pyrido[3,4-*b*]indoles or tetrahydro-β-carbolines (THβCs) are tricyclic compounds that are found in various natural sources that exhibit a wide range of important pharmacological activities. Chiral 1-substituted-THβCs, which have an asymmetric center at C1, have attained significant interest due to their possible Monoamine Oxidase (MAO) inhibitory activity, benzodiazepine receptor binding activity, and antimalarial effectiveness against chloroquine-resistant *Plasmodium falciparum*. This review highlights and summarizes various novel stereoselective approaches to introduce chirality at the C1 position of 1-substituted-THβCs in good yield and enantiomeric excess (ee) or diastereomeric excess (de). These methods include the Pictet–Spengler reaction, chiral auxiliary, Asymmetric Transfer Hydrogenation (ATH) with chiral catalysts, asymmetric addition reaction, and enzymatic catalysis. The syntheses of chiral THβCs are reviewed comprehensively, emphasizing their role in drug development from 1977 to 2024.

## Abstract 2

Chiral 1-substituted-THβC can be synthesized from l-tryptophan or tryptamine by Pictet–Spengler reaction and chiral auxiliary; also from substituted-DHβC by ATH reaction with chiral catalysts, asymmetric addition reaction, and enzymatic catalysis.

## Graphical abstract

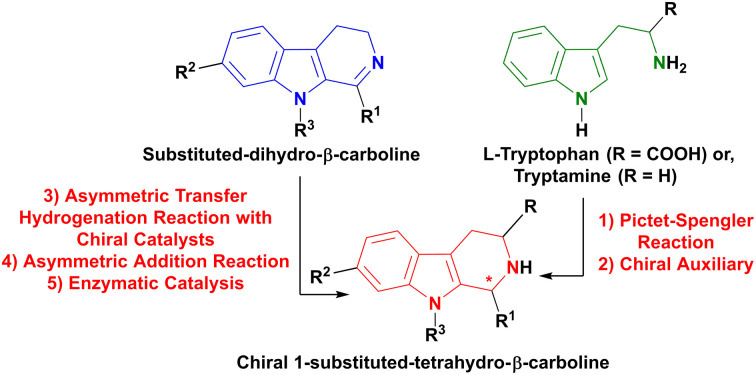

## 1. Introduction

**Fig. 1:**
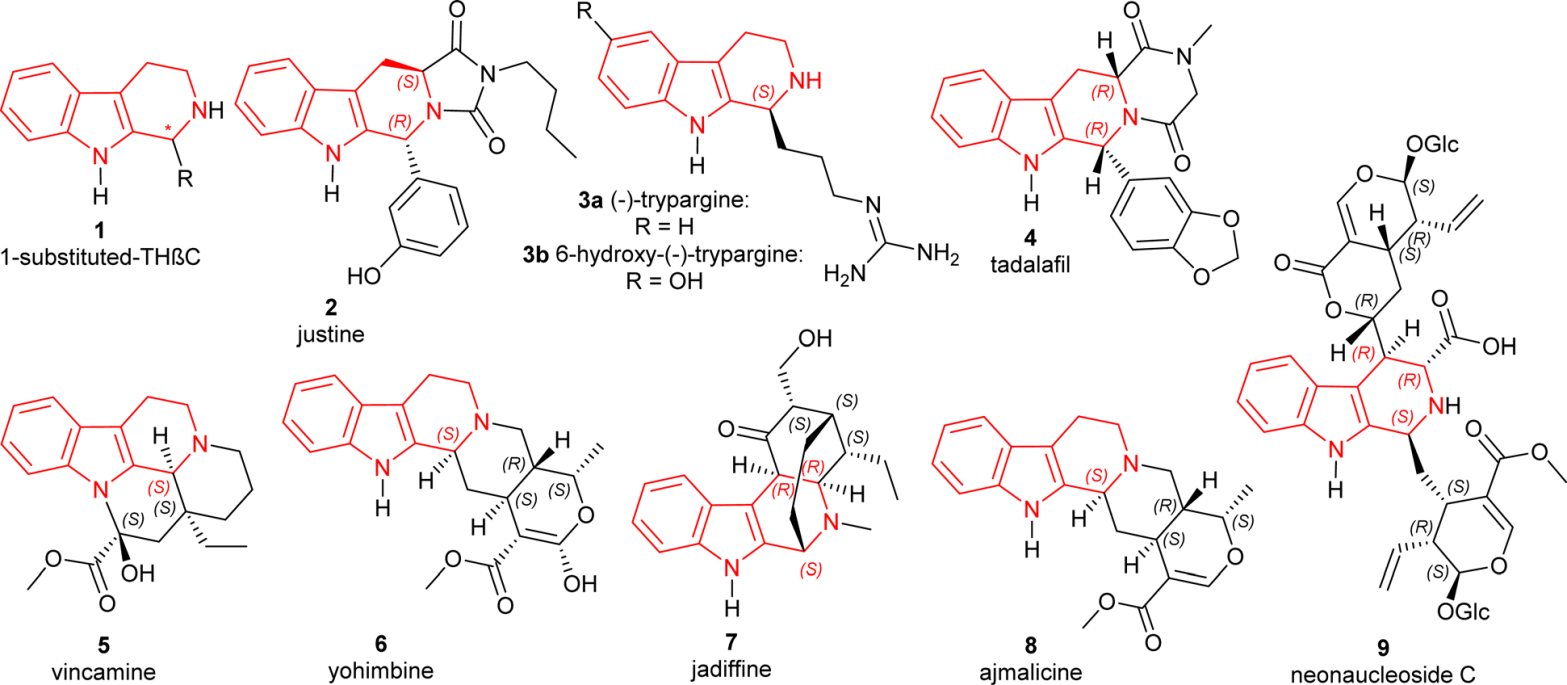
Structures of important chiral 1-substituted-THβCs.

The tetrahydro-β-carbolines (THβCs) are a group of compounds found in a variety of natural and synthetic compounds containing a unique tricyclic pyrido[3,4-*b*]indole ring and renowned for their promising biological actions. Originating from tryptamine or tryptophan, these compounds are widespread in nature and have been isolated from various sources including plants, fungi, animals, and marine organisms.^1^ THβCs exhibit a broad spectrum of pharmacological activities; including phosphodiesterase 5 (PDE5)-inhibitory,^2^ antitumor,^3,4^ antiviral,^5,6^ and antiprotozoal^7^ especially antimalarial effects.^8,9^ Chiral 1-substituted-THβCs 1 (Fig. 1), having an asymmetric center present at the C1 position, are still being sought even after being discovered more than a hundred years ago.^10^ They are mainly MAO inhibitors or work by binding to benzodiazepine receptors.^11,12^ They have gained particular interest due to their potential antimalarial efficacy against a *Plasmodium falciparum* strain (FcB1-Colombia) that is chloroquine-resistant.^13^

**Scheme 1:**
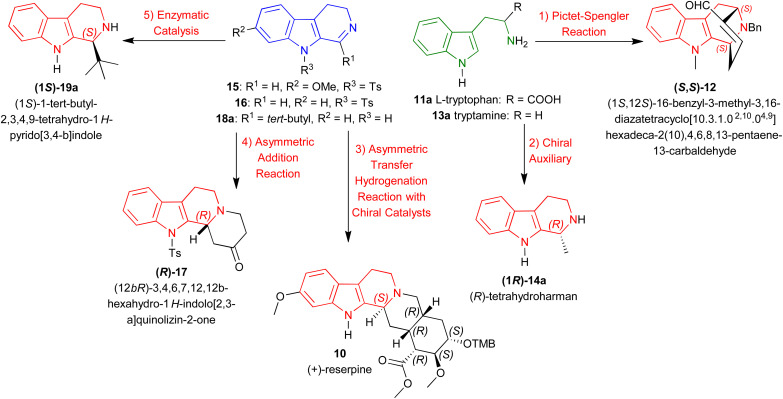
Representative examples of asymmetric methods for synthesizing chiral 1-substituted-tetrahydro-β-carbolines.

Some of the specific 1-substituted-THβCs (Fig. 1) that have biological importance are given below:

• Justine 2 (HR22C16) induces mitotic arrest and blocking cell division in taxol-resistant cancer cells.^4,14^

• The African rhacophorid frog *Kassina senegalensis*^15^ is the source of trypargine 3a, a highly poisonous THβC alkaloid. It was recently discovered in a hitherto unknown ground ascidian *Eudistoma* sp.^16^ A very similar chemical, 6-hydroxy-trypargine, was shown to be a strong neurotoxic in the venom of the Brazilian web spider *Parawixia bistriata*.^17^

• Tadalafil 4 is an orally active PDE5 inhibitor and also highly potent.^2,18^

• Vincamine 5 aided in mild to moderate dementia patients.^19^

• Yohimbine 6, an α_2_-adrenoceptor blocker that helps in erectile dysfunction.^20,21^

• Jadiffine 7 collected from *Vinca difformis*.^22^

• Ajmalicine 8 (ref. 23) and reserpine 10 (ref. 24) (Scheme 1) used as an antihypertensive.

• Neonaucleoside C 9 collected from *Neonauclea sessilifolia*.^25^

• Fumitremorgins are found in fungi that have antiviral^26^ and cell-cycle inhibitory activities.^27^ They also worked as protein kinase and topoisomerase II inhibitors.^28^

Synthetic methodologies to introduce chirality at the C1 position in THβCs have been extensively studied.^29^ These methods include the Pictet–Spengler reaction,^30^ asymmetric alkylation using N2-auxiliary as a directing group,^31^ and acid-induced epimerization in conjunction with the Pictet–Spengler reaction.^32^ Additionally, the Bischler–Napieralski reaction^33^ and classical Noyori ATH conditions^34^ have been highlighted as key synthetic routes to create chiral 1-substituted-THβCs. The C1 stereocenter in THβCs plays a crucial role in their pharmacological properties, influencing their activity in various therapeutic areas. With a ubiquitous presence in both natural sources and synthetic derivatives, these compounds have significant attention in medicinal chemistry for their potential therapeutic applications. The intricate interplay of their chemical structure and biological effects underscores their pivotal role in drug discovery endeavors, accentuating the paramount importance of advancing synthetic methodologies to access these compounds efficiently.

In the last 11 years, Laine *et al.*, Maity *et al.*, Szabó *et al.*, Wang *et al.*, and Du *et al.* published reviews that emphasized on the pharmacological importance, overall synthetic methods, biological activities, and applications of THβCs,^35–39^ but our review does not comprise any of the above-mentioned perspective wholeheartedly. This review neither talks about pharmacological importance, nor biological activities; neither gives all of the synthetic methodologies, nor the applications of THβCs also.

Instead, this review intends to offer a complete overview of the asymmetric synthesis of 1-substituted-THβCs, focusing on the synthetic methods to introduce chirality at the C1 position and their implications for drug development. Here, we discussed about five methods to create an asymmetric center at the C1 position of 1-substituted-THβCs reported from as early as 1977 to as latest as 2024. With representative examples (Scheme 1), they are:

(1) *Pictet–Spengler reaction*: From l-tryptophan 11a to synthesize (1*S*,12*S*)-16-benzyl-3-methyl-3,16-diazatetracyclo[10.3.1.0^2,10^.0^4,9^]hexadeca-2(10),4,6,8,13-pentaene-13-carbaldehyde (*S*,*S*)-12.^40^

(2) *Chiral auxiliary*: From tryptamine 13a to synthesize (*R*)-tetrahydroharman (1*R*)-14a.^41^

(3) *ATH with chiral catalysts*: From 7-methoxy-9-(4-methylphenyl)sulfonyl-3,4-dihydropyrido[3,4-*b*]indole (7-methoxy-9-tosyl-DHβC) 15 to synthesize (+)-reserpine 10.^42^

(4) *Asymmetric addition reaction*: From 9-tosyl-DHβC 16 to synthesize (12*bR*)-3,4,6,7,12,12*b*-hexahydro-1*H*-indolo[2,3-*a*]quinolizin-2-one (*R*)-17.^43^

(5) *Enzymatic catalysis*: From 1-*tert*-butyl-4,9-dihydro-3*H*-pyrido[3,4-*b*]indole 18a to synthesize (1*S*)-1-*tert*-butyl-THβC (1*S*)-19a.^44^

## 2. Enantioselective synthesis of 1-substituted-tetrahydro-β-carbolines

The enantioselective synthesis of 1-substituted-THβCs 1 can be conducted by the following five methods:

Method 1. Pictet–Spengler reaction.

Method 2. Chiral auxiliary.

Method 3. Asymmetric transfer hydrogenation reaction with chiral catalysts.

Method 4. Asymmetric addition reaction.

Method 5. Enzymatic catalysis.

### Method 1. Pictet–Spengler reaction

More than 113 years ago from now in 1911, Amé Pictet and Theodor Spengler devised a novel way to produce 1,2,3,4-tetrahydroisoquinoline by heating β-phenylethylamine and formaldehyde dimethylacetal in the presence of hydrochloric acid.^10^ This reaction is known as the Pictet–Spengler reaction. In 1928, Tatsui used the basis of this reaction to be the first to produce 1-methyl-THβC from tryptamine and ethanal in the presence of sulphuric acid.^45^

#### Example 1. Asymmetric formal syntheses of (−)-koumine, (−)-taberpsychine, and (−)-koumidine intermediates from l-tryptophan

Bailey and McLay asymmetrically synthesized intermediates of naturally occurring (+)-koumine,^46^ (+)-taberpsychine^47^ & (+)-koumidine.^46,48^

First, l-tryptophan methyl ester 11b was condensed with methyl 4-oxobutanoate at 0 °C with excess 2,2,2-trifluoroacetic acid (TFA) in dichloromethane (DCM) to get (1*S*,3*S*)-20 (predominating than its (1*R*,3*S*)-diastereomer by 4 : 1 diastereomeric ratio or dr) by Pictet–Spengler reaction under kinetic control^49^ with a total yield of 61%.

The N2 of (1*S*,3*S*)-20 was then protected by benzyl carbonochloridate producing 21, and N9 of 21 was protected by methyl iodide (CH_3_I)/sodium hydride (NaH) or benzyl iodide/NaH respectively at 0 °C giving 22a or 22b.

With NaH and protic methanol (MeOH), Dieckmann cyclization of 22a and 22b gave the β-keto ester 23a and 23b and their enolic form 24a and 24b. These esters were hydrolyzed and decarboxylated by heating at 130 °C with NaCl and H_2_O in *N*,*N*-dimethylformamide (DMF)^50^ producing the bridged ketone 25a (>95% ee) and 25b.

25a was then reacted with Tf_2_NPh/NaH/THF, and LiCN/benzene (PhH)/Pd-(PPh_3_)_4_ (ref. 51) to get benzyl (1*S*,12*S*)-13-hydroxy-3-methyl-3,16-diazatetracyclo[10.3.1.0^2,10^.0^4,9^]hexadeca-2(10),4,6,8,13-pentaene-16-carboxylate 26 which possesses an α,β-unsaturated nitrile for Michael addition of a C_4_ fragment, giving access to the full carbon skeleton of N9-methylated alkaloids of the ajmaline–sarpagine group. This overall route is more efficient than that of the N2-benzyl derivative of 26.^52^

**Scheme 2:**
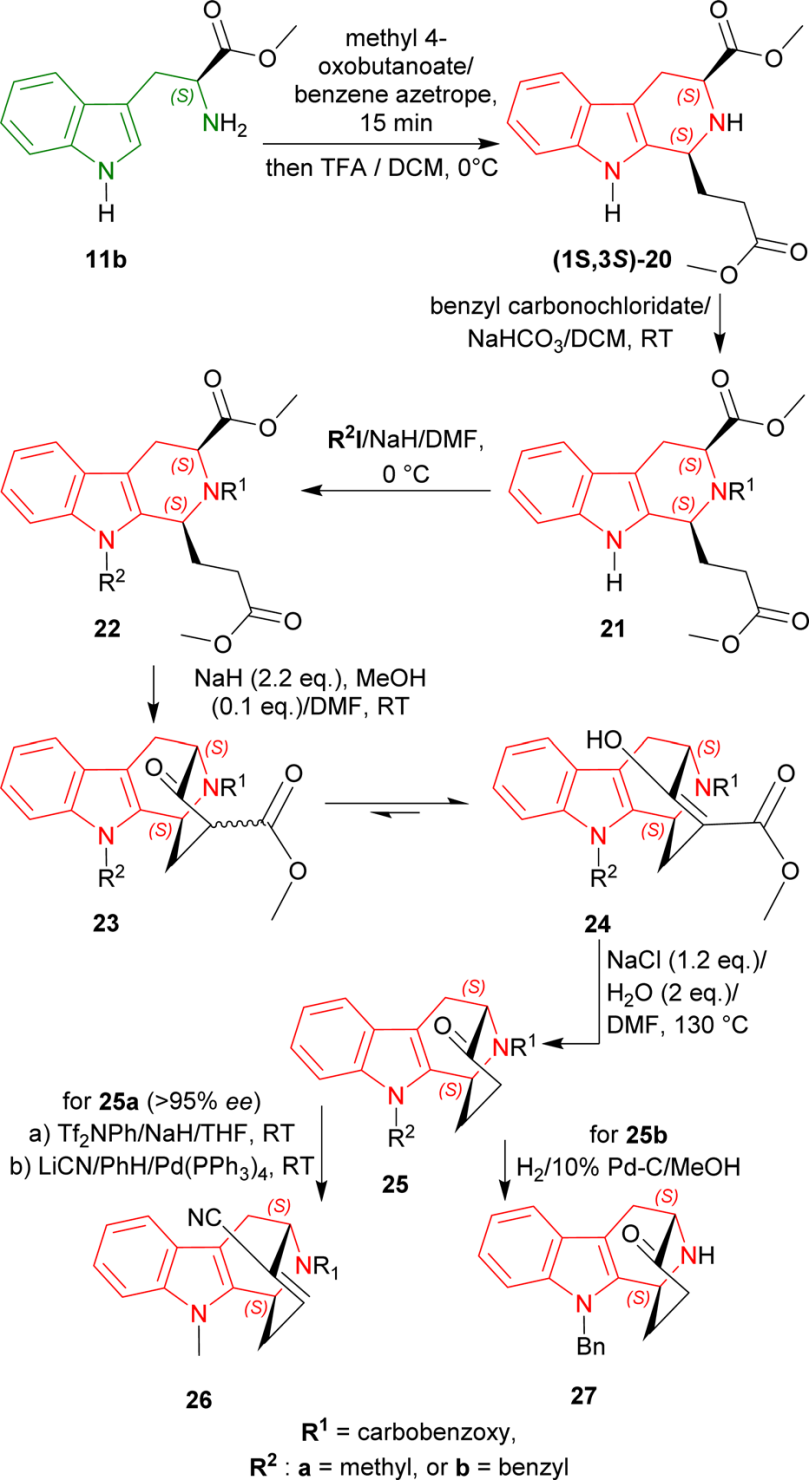
Asymmetric formal syntheses of (−)-koumine, (−)-taberpsychine, and (−)-koumidine intermediates from l-tryptophan methyl ester.

On the other hand, catalytic hydrogenation of 25b with 10% Pd–C in MeOH produced (1*S*,12*S*)-3-benzyl-13-oxo-3,16-diazatetracyclo[10.3.1.0^2,10^.0^4,9^]hexadeca-2(10),4,6,8-tetraene 27 which is the antipode of the intermediate used in the syntheses of (+)-koumine, (+)-taberpsychine, and (+)-koumidine (Scheme 2).^53^

So, methyl 4-oxobutanoate predominantly produced (1*S*,3*S*)-diastereomer with l-tryptophan methyl ester by Pictet–Spengler reaction under kinetic control.

#### Example 2. Modified Pictet–Spengler reaction for formal syntheses of (−)-suaveoline, (−)-raumacline, and (−)-*N*^b^-methylraumacline intermediates

Bailey *et al.* devised reaction pathways to produce intermediates of the ajmaline–sarpagine family alkaloids such as (−)-suaveoline, (−)-raumacline, and (−)-*N*^b^-methylraumacline.^40^

l-Tryptophan 11a was converted to its homologated nitrile 28 in four steps in 50% overall yield.^54^ Modified Pictet–Spengler reaction of 28 with methyl prop-2-ynoate followed by treatment with TFA gave rise to a 60% yield of the acetate (1*S*,3*S*)-29 (54% de). In this reaction, Bailey *et al.* used a carbonyl-conjugated alkyne instead of the conventional aldehyde.^52,55,56^

**Scheme 3:**
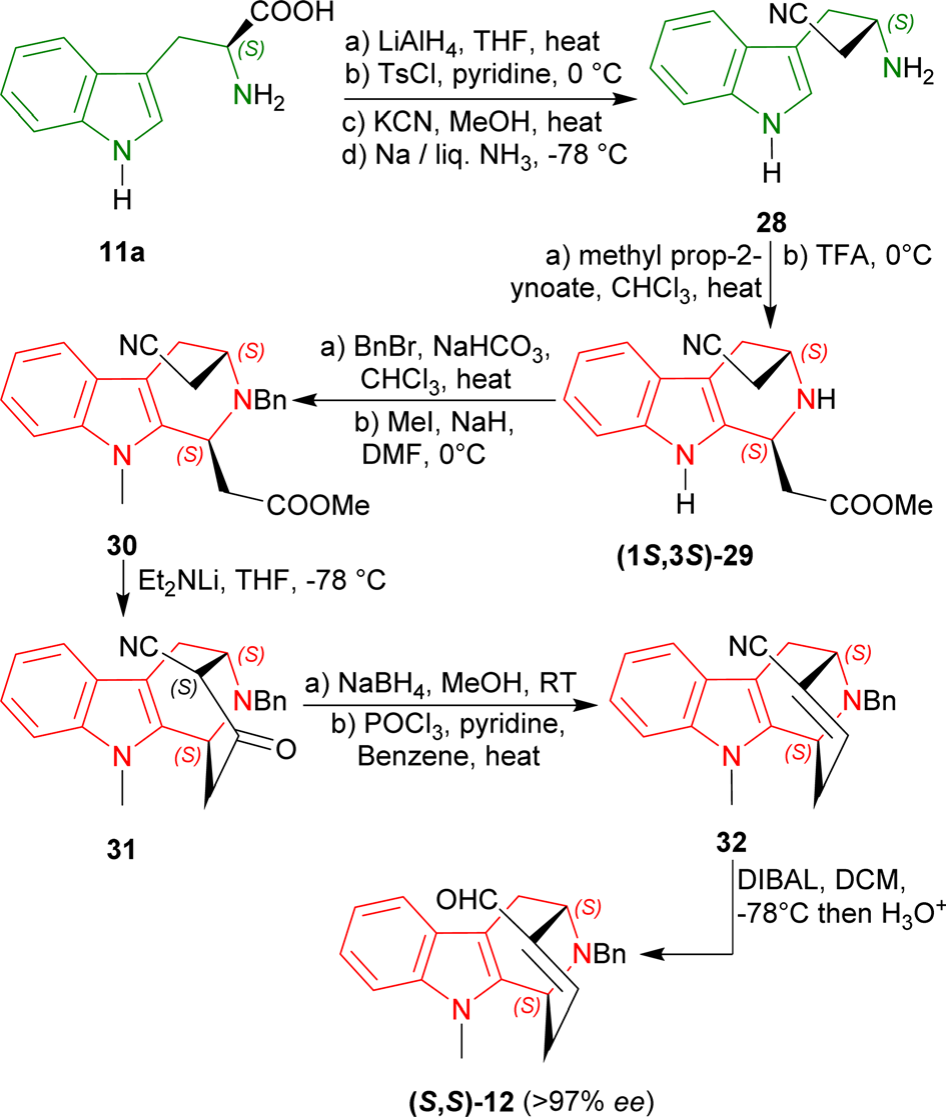
Asymmetric formal syntheses of (−)-suaveoline, (−)-raumacline, and (−)-*N*^b^-methylraumacline intermediates from l-tryptophan.

N2 benzylation and N9 methylation of (1*S*,3*S*)-29 furnished the compound 30 in an overall 46% total yield. With lithium diethylamide at −78 °C, Dieckmann/Thorpe cyclisation^57^ of 30 gave 31 in 90% yield. The reduction of 31 with sodium borohydride in MeOH at room temperature (RT) afforded the corresponding hydroxynitrile^58^ and dehydration with POCl_3_ produced 32 in 87% yield. Finally, reduction with bis(2-methylpropyl)alumane (DIBAL) gave a 99% yield of (*S*,*S*)-12 (>97% ee) which was used in the synthesis of (−)-suaveoline, (−)-raumacline and (−)-*N*^b^-methylraumacline (Scheme 3).^59^

So, methyl prop-2-ynoate predominantly produced (1*S*,3*S*)-diastereomer with (3*S*)-3-amino-4-(1*H*-indol-3-yl)butanenitrile by modified Pictet–Spengler reaction.

#### Example 3. Catalytic asymmetric Pictet–Spengler reaction with chiral organic Brønsted acid

**Scheme 4:**
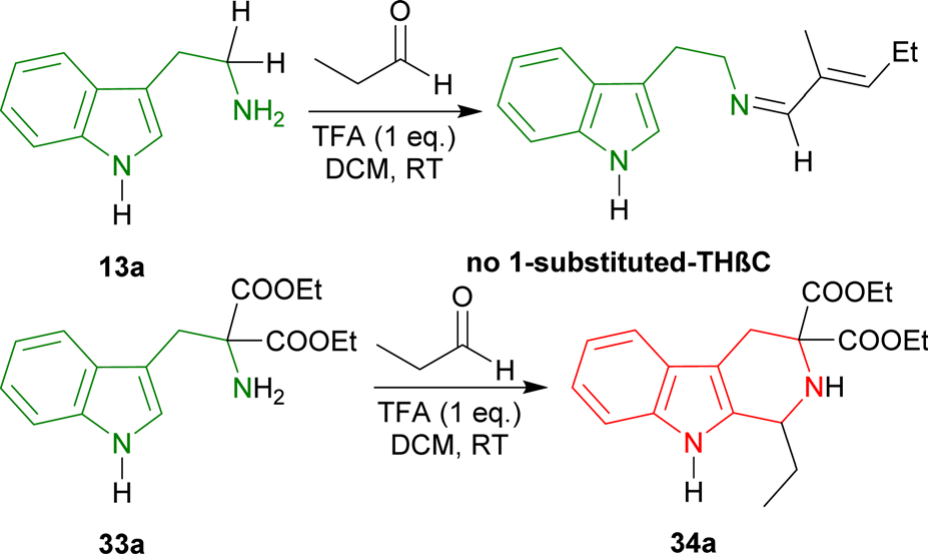
Tryptamine failed to produce any 1-substituted-THβC by Pictet–Spengler reaction but diethyl 2-amino-2-(1*H*-indol-3-ylmethyl)propanedioate was able to produce diethyl 1-ethyl-1,2,4,9-tetrahydropyrido[3,4-*b*]indole-3,3-dicarboxylate.

Seayad *et al.* experimented on the acid catalysis of the Pictet–Spengler reaction. Still, they failed to cyclize tryptamine 13a to produce any 1-substituted-THβC in the presence of propanal and TFA in DCM at RT, but diethyl 2-amino-2-(1*H*-indol-3-ylmethyl)propanedioate 33a gave >90% yield of diethyl 1-ethyl-THβC-3,3-dicarboxylate 34a (Scheme 4).^60^

**Fig. 2:**
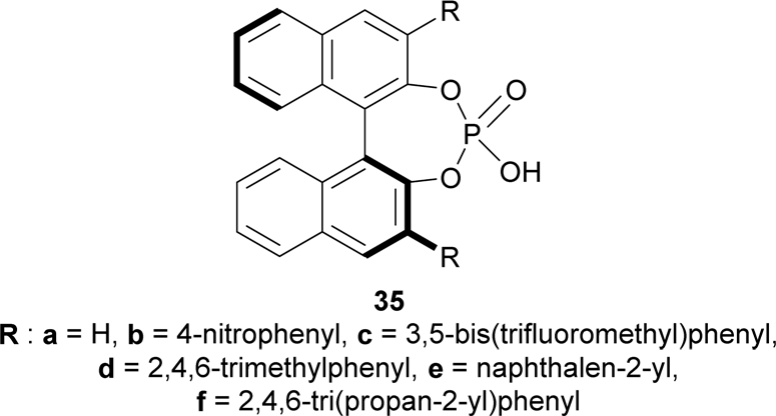
Chiral organic Brønsted acid for catalytic asymmetric Pictet–Spengler reaction.

**Scheme 5:**
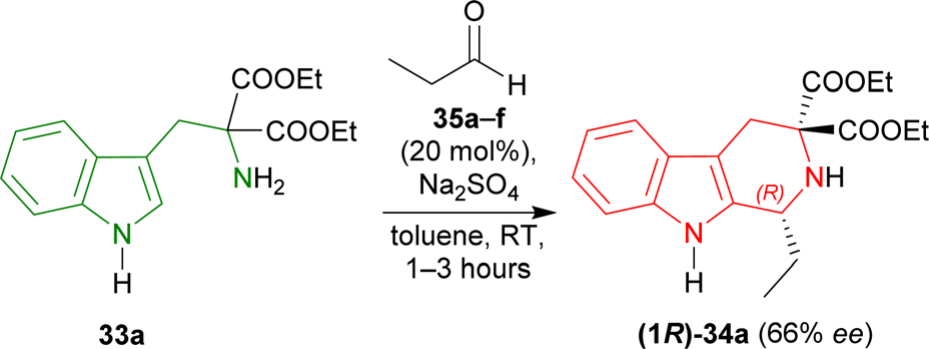
Screening of chiral organic Brønsted acid for catalytic asymmetric Pictet–Spengler reaction.

To find an appropriate chiral organic Brønsted acid, 20 mol% 35a–f (Fig. 2) was then examined with Na_2_SO_4_ in toluene at RT for 1–3 hours. Among them, 35f gave the highest 66% ee with a good yield of 90% of (1*R*)-34a (Scheme 5).

**Scheme 6:**
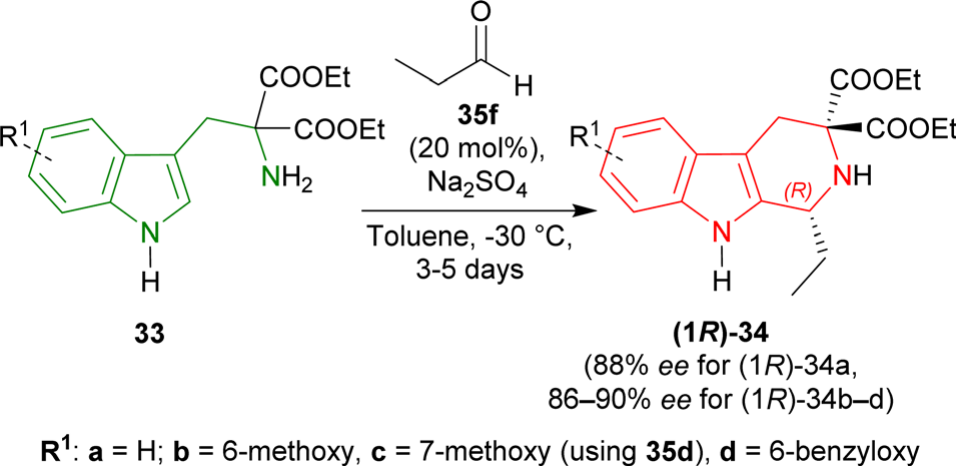
Screening of diethyl 2-amino-2-(1*H*-indol-3-ylmethyl)propanedioate derivatives.

When the previous reaction was conducted with 35f at −30 °C for 3–5 days, the yield of (1*R*)-34a decreased to 76% while ee increased to 88%. 33b–d gave a similar ee of 86–90% with an excellent yield of 94–98% for (1*R*)-34b–d (Scheme 6).

**Scheme 7:**
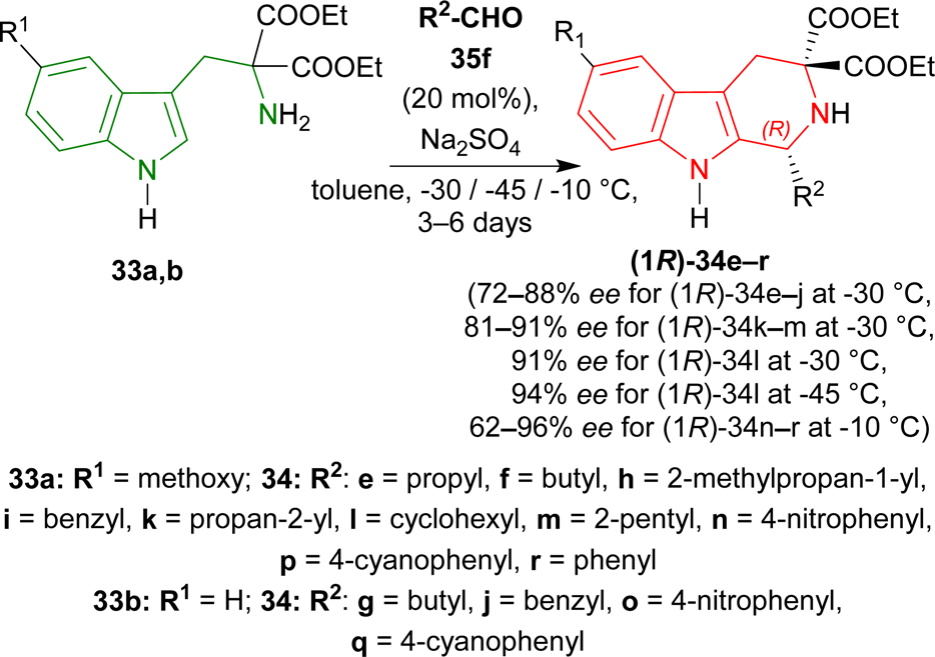
Screening of aldehydes.

Various aldehydes were then reacted with 33a,b. Aliphatic unbranched and branched aldehydes produced (1*R*)-34e–j (58–98% yield, 72–88% ee) and (1*R*)-34k–m (50–93% yield, 81–91% ee) respectively at −30 °C in toluene for 3–6 days. When the temperature was decreased from −30 °C to −45 °C, ee of (1*R*)-34l increased slightly from 91% to 94% but yield decreased from 93% to 64%. Aromatic and electron-poor aromatic aldehydes also gave moderate to good yield (40–98%) and ee (62–96%) for (1*R*)-34n–r at −10 °C in DCM (Scheme 7).

So, propionaldehyde and other aldehydes predominantly produced (1*R*)-enantiomer with diethyl 2-amino-2-(1*H*-indol-3-ylmethyl)propanedioate and its derivatives catalyzed by chiral organic Brønsted acid.

#### Example 4. Synthesis of (1*S*,3*S*,4*R*)-THβCs from 1*H*-indole through Friedel–Crafts/Henry adducts

Arai *et al.* developed a four-step synthetic pathway to produce chiral 1-substituted-THβCs from 1*H*-indole.^61^

**Scheme 8:**
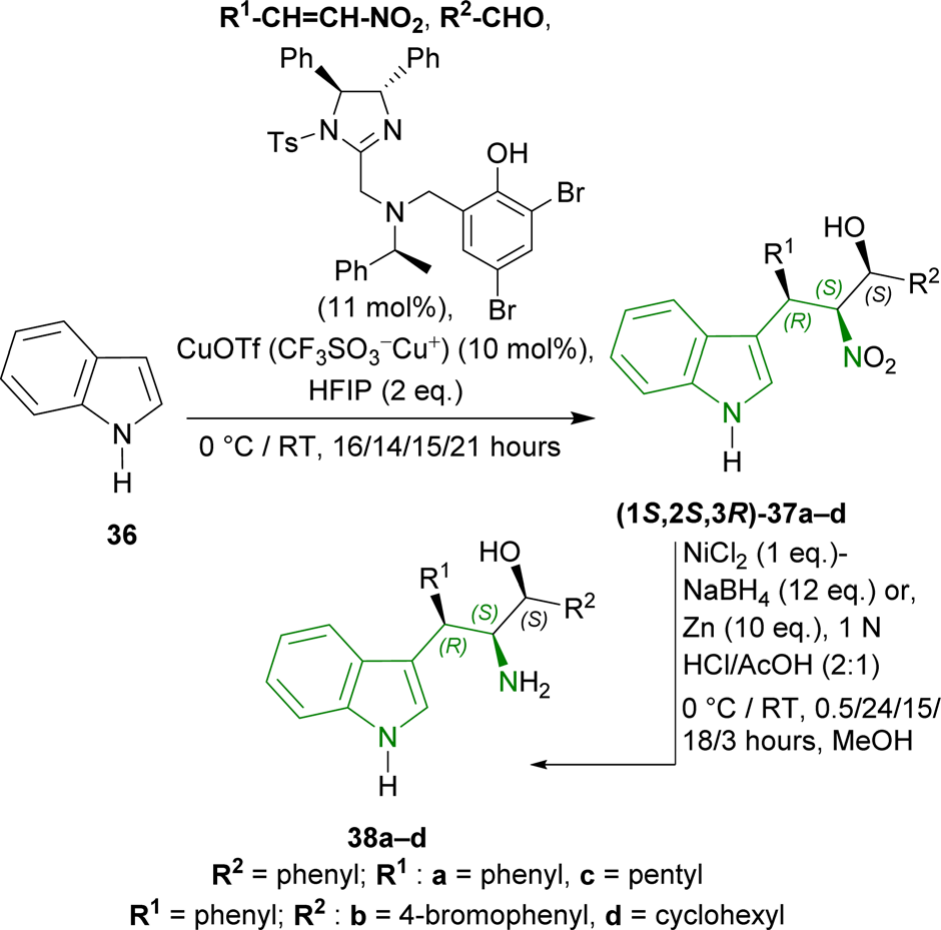
Synthesizing Friedel–Crafts/Henry adducts from 1*H*-indole and their reduction.

1*H*-indole 36 was reacted with different nitroalkenes and aldehydes in the presence of 2,4-dibromo-6-[[[(4*S*,5*S*)-1-(4-methylphenyl)sulfonyl-4,5-diphenyl-4,5-dihydroImidazol-2-yl]methyl-[(1*S*)-1-phenylethyl]amino]methyl]phenol (11 mol%) (Scheme 8), copper(i) trifluoromethanesulfonate (CuOTf or CF_3_SO_3_^−^Cu^+^, 10 mol%), fluoro(iodo)phosphane (HFIP, 2 equivalents or eq.)^62^ in toluene to produce (1*S*,2*S*,3*R*)-37a–d in 76–84% yield and 97–99% ee at 0 °C or RT which are (*R*,*S*,*S*)-Friedel–Crafts/Henry adducts. Next (1*S*,2*S*,3*R*)-37a, reduced with nickel boride,^63,64^ gave (1*S*,2*S*,3*R*)-3-(1*H*-indol-3-yl)-2-amino-1,3-diphenylpropan-1-ol 38a (20% yield) at 0 °C for 0.5 hour. But, Zn powder under acidic condition^65^ at 0 °C for 24 hours gave a 59% yield of 38a. At RT for 15–18 hours, this condition gave a 99% yield of 38a–b from (1*S*,2*S*,3*R*)-37a–b. Ultimately, Zn-nanopowder was used to reduce the reaction time to 3 hours at RT to give 98–99% yields of 38c–d from (1*S*,2*S*,3*R*)-37c–d (Scheme 8).

**Scheme 9:**
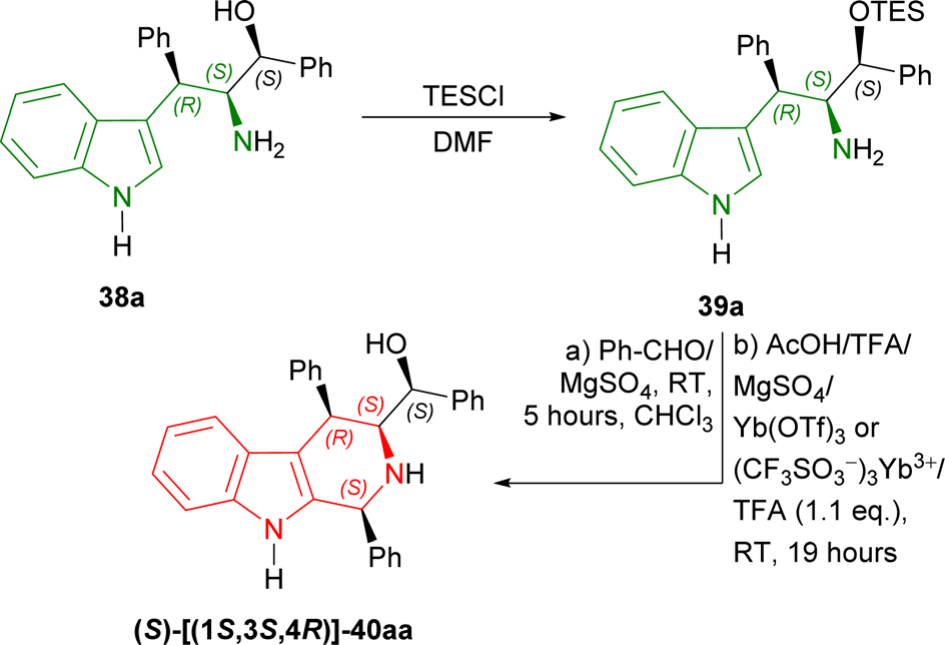
Protecting the OH group with TES group, Pictet–Spengler reaction, and removing the OH-protection.

Then, 38a was experimented with to optimize Pictet–Spengler reaction. 38a was converted into 39a with triethylsilyl chloride (TESCl) in DMF. 39a was cyclized with benzaldehyde and ethanoic acid, TFA, MgSO_4_, ytterbium(iii) trifluoromethanesulfonate (Yb(OTf)_3_ or (CF_3_SO_3_^−^)_3_Yb^3+^); but TFA gave 30% yield of (*S*)-[(1*S*,3*S*,4*R*)-1,4-diphenyl-2,3,4,9-tetrahydro-1*H*-pyrido[3,4-*b*]indol-3-yl]-phenylmethanol 40aa. After that, 39a with TFA (1.1 eq.) at RT for 19 hours following without and with MgSO_4_ at RT for 5 hours in CHCl_3_ gave 47% and 67% yield of 40aa respectively (Scheme 9).

**Scheme 10:**
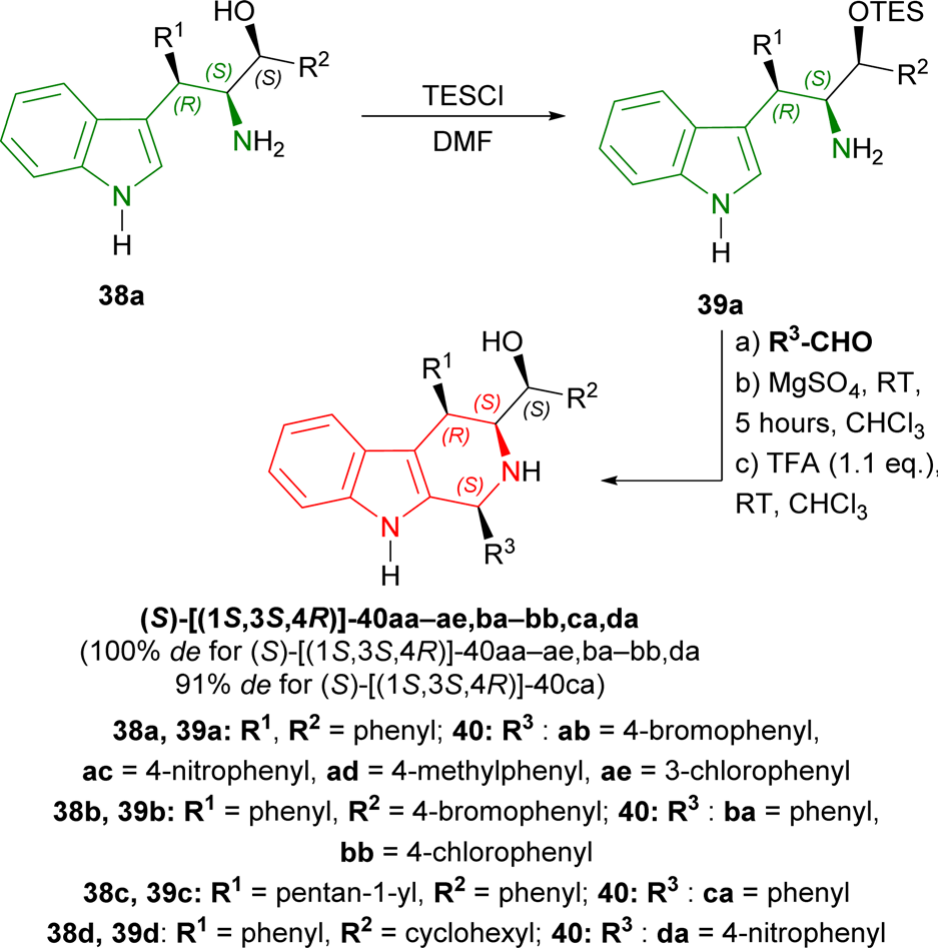
Screening of aldehydes.

38a–d was tested with different aromatic aldehydes for 19–25 hours to give (*S*)-[(1*S*,3*S*,4*R*)]-40ab–ae,ba–bb,ca,da in 38–72% yields. Every product had 100% de except (*S*)-[(1*S*,3*S*,4*R*)]-40ca of which de was 91% (Scheme 10).

So, phenylaldehyde and other aldehydes produced (1*S*,3*S*,4*R*)-THβCs predominantly with (1*S*,2*S*,3*R*)-1-(1*H*-indol-3-yl)-1,3-diphenyl-3-triethylsilyloxypropan-2-amine and its derivatives (made from Friedel–Crafts/Henry adducts) by Pictet–Spengler reaction.

#### Example 5. Two methods for TFA-catalyzed Pictet–Spengler reaction

Vavsari *et al.* developed two methods for TFA-catalyzed Pictet–Spengler reaction.^66^

**Scheme 11:**
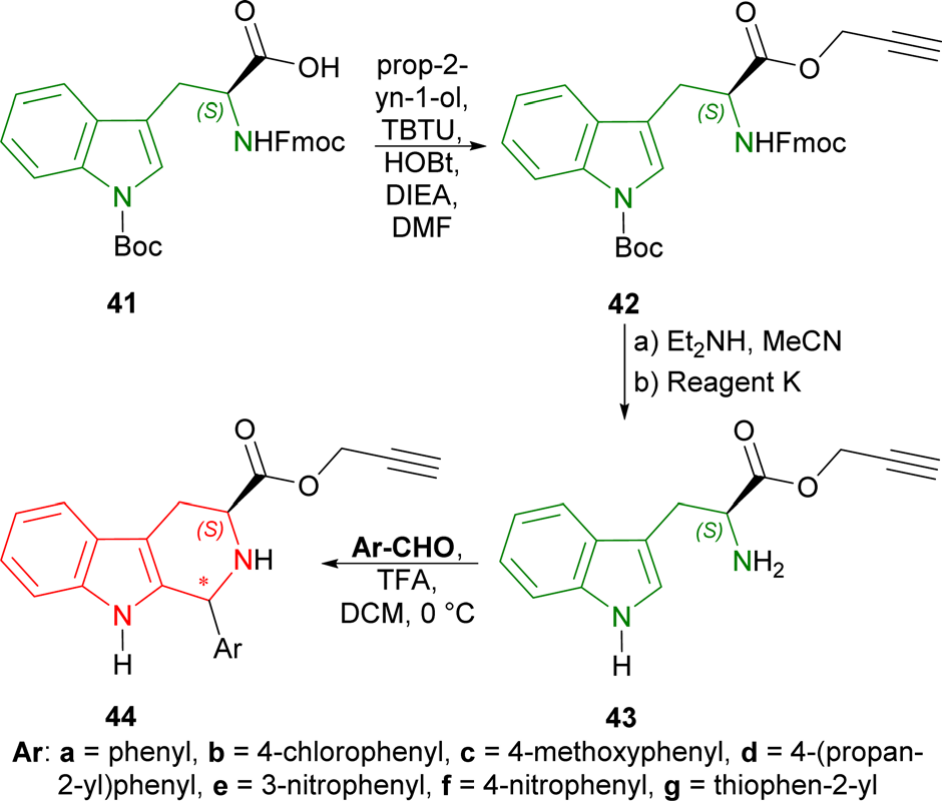
Synthesizing prop-2-ynyl ester of (2*S*)-2-(9*H*-fluoren-9-ylmethoxycarbonylamino)-3-[1-[(2-methylpropan-2-yl)oxycarbonyl]indol-3-yl]propanoic acid, removing Fmoc protection group, followed by Pictet–Spengler reaction.

Firstly, (2*S*)-2-(9*H*-fluoren-9-ylmethoxycarbonylamino)-3-[1-[(2-methylpropan-2-yl)oxycarbonyl]indol-3-yl]propanoic acid 41 (10 mmol) was treated with prop-2-yn-1-ol (2 eq.), [benzotriazol-1-yloxy(dimethylamino)methylidene]-dimethylazanium;tetrafluoroborate (TBTU, 1.1 eq.), 1-hydroxybenzotriazole (HOBt·H_2_O, 1.1 eq.), *N*-ethyl-*N*-propan-2-ylpropan-2-amine (DIEA, 2.2 eq.) and in DMF to get 87% yield of 42. The Fmoc protection was then removed by diethylamine and acetonitrile and the Boc protection group was eliminated by cooled reagent K (TFA, water, phenol, ethanedithiol, triethylsilane, thioanisol) gaining a 66% yield of 43. The compound 43 was then reacted with various aromatic aldehydes and TFA in DCM at 0 °C. Benzaldehyde gave higher yield (73% for prop-2-ynyl (1*S*,3*S*)-1-phenyl-THβC-3-carboxylate (1*S*,3*S*)-44a) than 3- and 4-substituted-benzaldehydes (52–67% yields for prop-2-ynyl (1*S*,3*S*)-1-substituted-THβC-3-carboxylate (1*S*,3*S*)-44b–f) and thiophene-2-carbaldehyde (57% yield for prop-2-ynyl (1*R*,3*S*)-1-thiophen-2-yl-THβC-3-carboxylate (1*R*,3*S*)-44g) (Scheme 11).

**Scheme 12:**
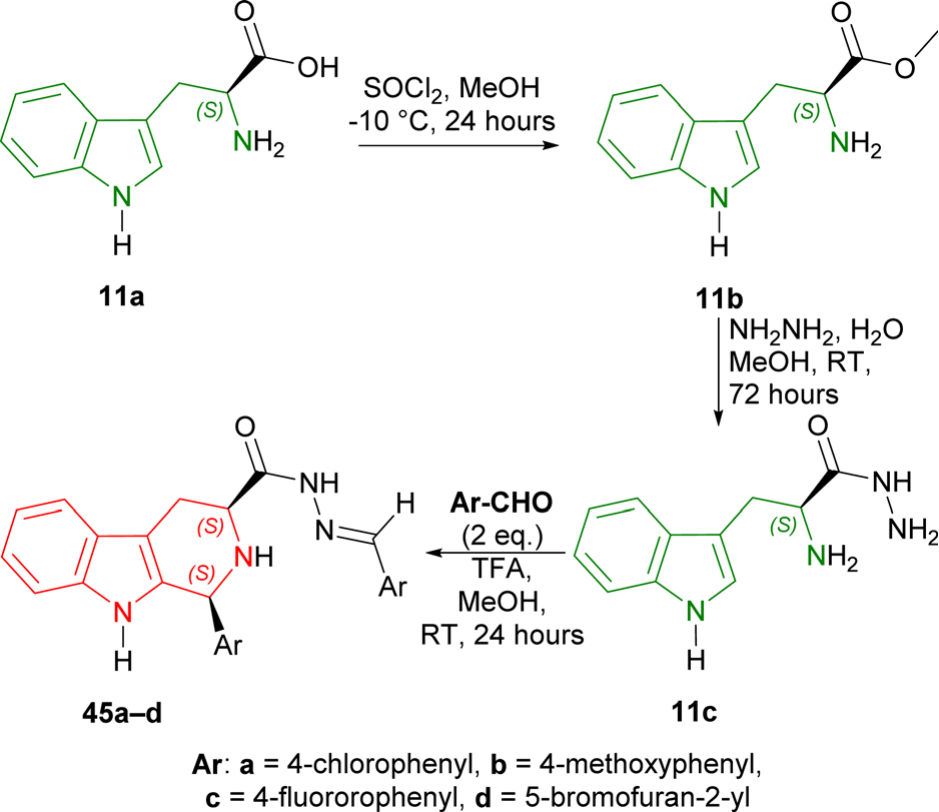
Synthesizing methyl ester of l-tryptophan, then l-tryptophan hydrazide, and Pictet–Spengler reaction.

Secondly, l-tryptophan 11a is converted to 11b (95% yield) with thionyl chloride in MeOH at −10 °C for 24 hours. With 11b and hydrazine in MeOH at RT for 72 hours, 11c was obtained in 95% yield. 11c was then reacted with aromatic aldehydes in the presence of TFA as a catalyst in MeOH at RT for 24 hours. 4-Substituted-benzaldehydes gave similar yields (78–83% for (1*S*,3*S*)-1-(4-substituted-phenyl)-*N*-[(*E*)-(4-substituted-phenyl)methylideneamino]-THβC-3-carboxamide 45a–c); while 5-bromofuran-2-aldehyde had slightly better yield (85% for (1*S*,3*S*)-1-(5-bromofuran-2-yl)-*N*-[(*E*)-(5-bromofuran-2-yl)methylideneamino]-THβC-3-carboxamide 45d) (Scheme 12).

So, prop-2-ynyl (2*S*)-2-amino-3-(1*H*-indol-3-yl)propanoate and l-tryptophan hydrazide produced mainly (1*S*,3*S*)-THβCs with aromatic aldehydes by TFA-catalyzed Pictet–Spengler reaction; the only exception being prop-2-ynyl (1*R*,3*S*)-1-thiophen-2-yl-THβC-3-carboxylate.

### Method 2. Chiral auxiliary

A chiral auxiliary is actually a pure enantiomeric organic chemical that is coupled with the starting material to generate a new product that may then undergo diastereoselective reactions using intramolecular asymmetric induction.^67,68^ At the end of the reaction, the auxiliary is removed under circumstances that ensures no racemization of the product. Then, it is often recovered and reused. Two of the widely used chiral auxiliaries are: Evans oxazolidinones,^69^ and Oppolzer sultams.^70^ There are many applications with the use of chiral auxiliaries.^67,71^

#### Example 1. Asymmetric synthesis of (1*S*)-1-methyl-THβC and (1*S*)-1-phenyl-THβC with (2*R*)-2-amino-2-phenylethanol as a chiral auxiliary

Qais *et al.* synthesized (1*S*)-1-methyl-THβC and (1*S*)-1-phenyl-THβC with the help of (2*R*)-2-amino-2-phenylethanol as a chiral auxiliary.^72^

**Scheme 13:**
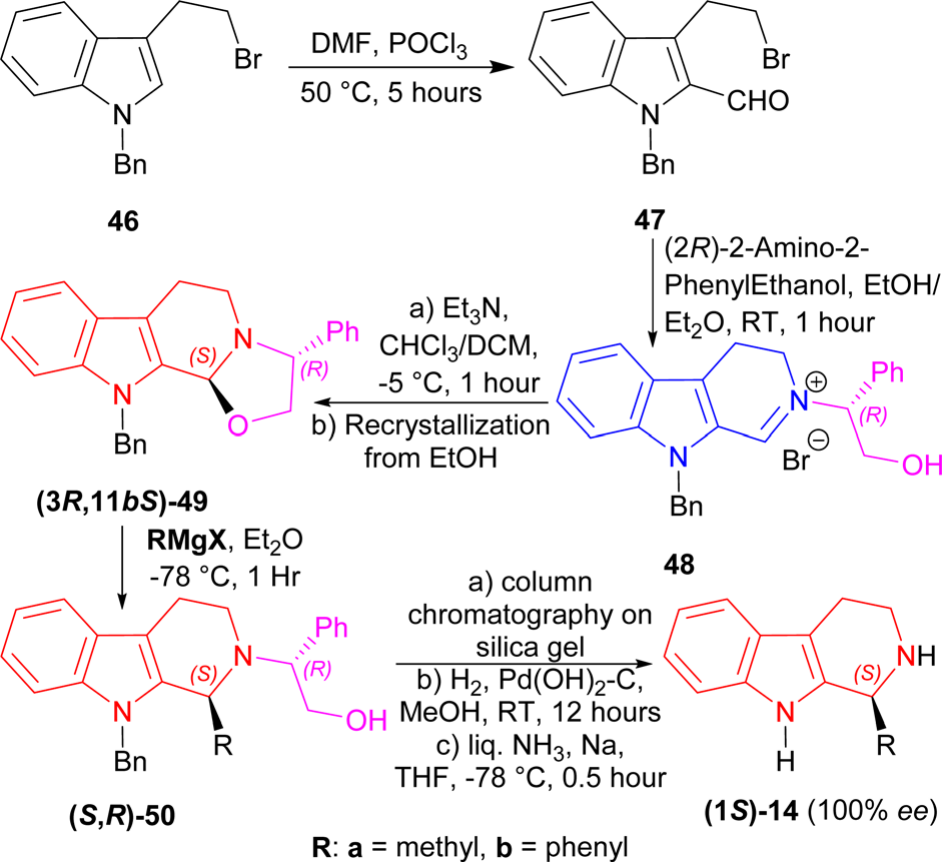
Synthesizing (1*S*)-1-methyl-THβC and (1*S*)-1-phenyl-THβC with (2*R*)-2-amino-2-phenylethanol as chiral auxiliary.

1-Benzyl-3-(2-bromoethyl)indole 46 underwent Vilsmeier–Haack reaction to get 47 (50% yield). With (2*R*)-2-amino-2-phenylethanol at RT for 1 hour, 47 formed the iminium salt 48 after azeotropic distillation with benzene. Treatment with triethylamine (Et_3_N) at −5 °C for 1 hour in chloroform/DCM then cyclized 48 into (3*R*,11*bS*)-49 (85% de); after recrystallization from ethanol, it was found in 100% de with 62% yield. Then, (3*R*,11*bS*)-49 was reacted with two Grignard reagents (MeMgI and PhMgI) at −78 °C for 1 hour to give 90% de of (*S*,*R*)-50a and (*S*,*R*)-50b. The purification process involved column chromatography on silica gel. Hydrogenolysis on Pd(OH)_2_–carbon at RT for 12 hours will remove the chiral auxiliary, and sodium in liquid ammonia removed the *N*-benzyl group with 100% ee of (1*S*)-1-methyl-THβC (1*S*)-14a and (1*S*)-1-phenyl-THβC (1*S*)-14b (Scheme 13).

So, the (*R*)-configured chiral auxiliary (2*R*)-2-amino-2-phenylethanol predominantly produced (1*S*)-1-substituted-THβCs.

#### Example 2. Enantioselective Synthesis of (*R*)-tetrahydroharman with chiral acetylenic sulfoxides as chiral auxiliaries

Lee *et al.* used two chiral acetylenic sulfoxides, 1-[(*R*)-ethynylsulfinyl]-2-nitrobenzene (*R*)-51a and 1-[(*R*)-ethynylsulfinyl]-4-methylbenzene (*R*)-51b, to enantioselectively synthesize (*R*)-tetrahydroharman by Michael addition and cyclization.^41^

**Scheme 14:**
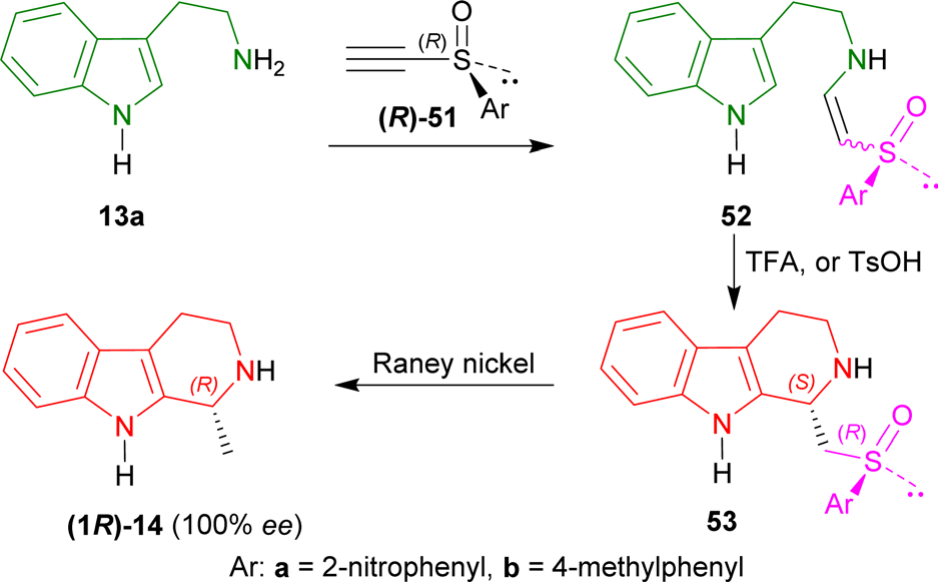
Synthesizing (*R*)-tetrahydroharman with chiral acetylenic sulfoxides as chiral auxiliary.

At first, tryptamine 13a was added with (*R*)-51a,b to form 52a and 52b. Then 52a,b was cyclized with TFA or toluene-*p*-sulfonic acid (*p*-TsOH) to form 53a and 53b as a major compound. RANEY® nickel desulfurization of 53a,b then resulted in 80% yield of optically pure (100% ee) (*R*)-tetrahydroharman (1*R*)-14 (ref. 73 and 74) (overall 57% yield) (Scheme 14).

So, (*R*)-configurated chiral acetylenic sulfoxides produced (*R*)-configurated THβC named (*R*)-tetrahydroharman.

#### Example 3. Asymmetric synthesis of 1-substituted-THβC using pyroglutamic acid derivatives as chiral auxiliaries

Itoh *et al.* used (*S*)-pyroglutamic acid derivatives as chiral auxiliaries to synthesize 1-substituted-THβC.^75^

**Scheme 15:**
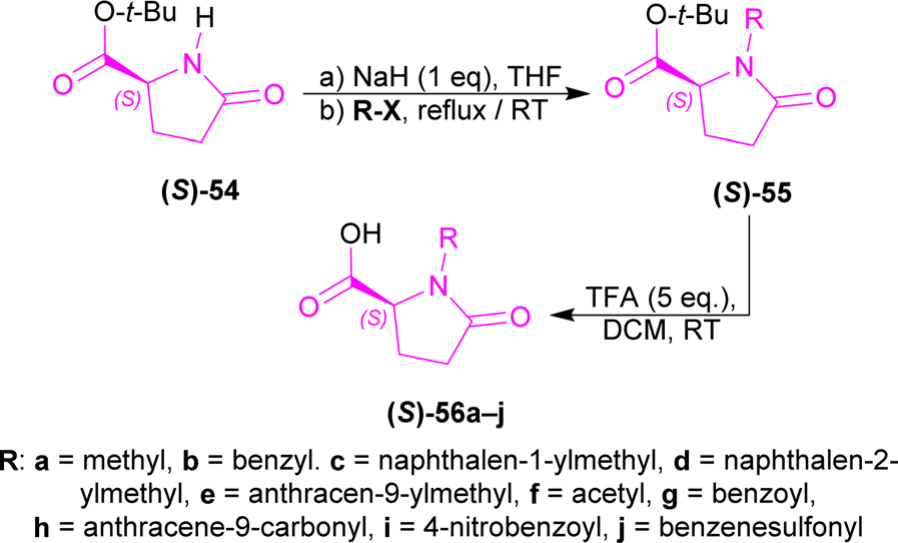
Synthesis of (*S*)-pyroglutamic acid derivatives.

*tert*-Butyl (*S*)-pyroglutamate (*S*)-54, NaH with various *N*-protecting reagents (R-X) producing (*S*)-55a–j (highest 94% yield for (*S*)-55a); which was converted to (2*S*)-1-substituted-5-oxopyrrolidine-2-carboxylic acid (*S*)-56a–j (highest 97% yield for (2*S*)-1-(2-naphthylmethyl)-5-oxopyrrolidine-2-carboxylic acid (*S*)-56d) using TFA at RT (Scheme 15).

(*S*)-56a–j were reacted with β-carboline 57 and 1-(3-dimethylaminopropyl)-3-ethylcarbodiimide hydrochloride (EDCI) to get 58a–j. Among these, 58g reached 99% yield in 20 hours but, 58e reached 95% yield in 4 hours.

58a–j was reacted with 2,2,2-trichloroethylcarbonyl chloride (2 eq.), and tributyl(prop-2-enyl)stannane (allyltributyltin, 3 eq.) as a nucleophile in DCM at −40 °C for 24 hours to produce 59a–j. Among these, 59d,f,i were found in quantitative yields; and 59e,b,g,c in good yields of 98, 95, 92, and 87%. NaOH in THF-H_2_O at RT for 1.5–2.5 hours were needed to remove the chiral auxiliary to give 2,2,2-trichloroethyl 1-prop-2-enyl-1,9-dihydropyrido[3,4-*b*]indole-2-carboxylate 60.

**Scheme 16:**
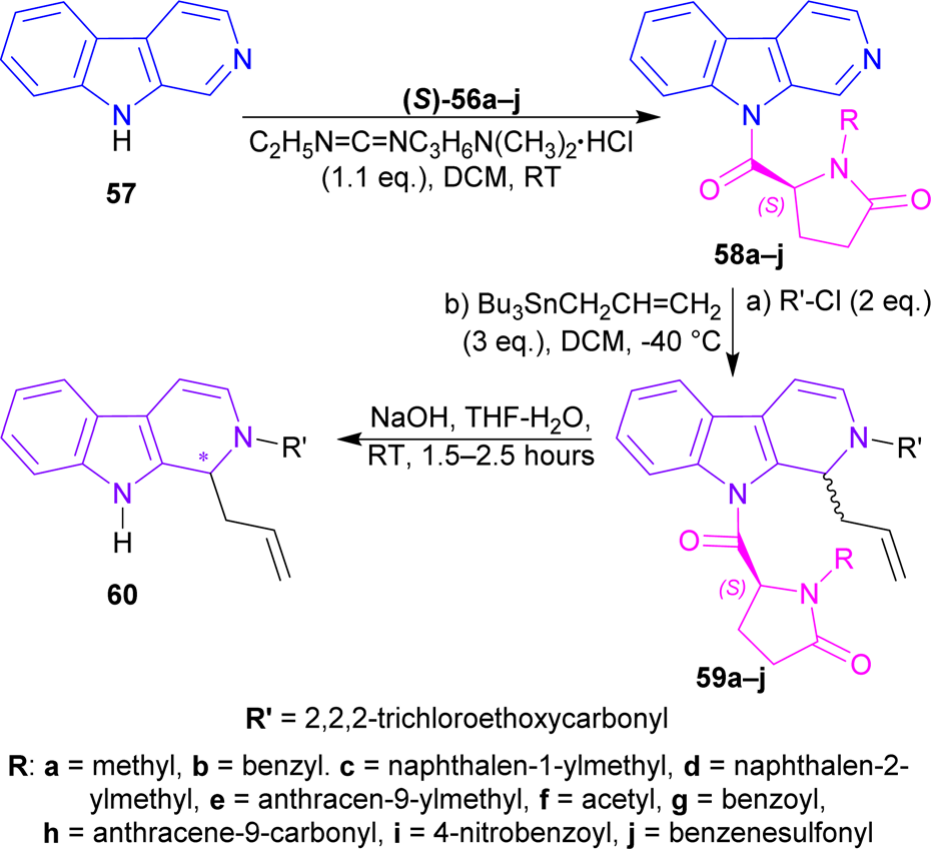
N9 addition of chiral auxiliary to the β-carboline, C1 addition of allyltributyltin and N2 protecting, then ultimately removal of the chiral auxiliary.

For 59a–e, which had alkyl *N*-protecting groups, (*S*)-configuration product (1*S*)-2,2,2-trichloroethyl 1-prop-2-enyl-1,9-dihydropyrido[3,4-*b*]indole-2-carboxylate (1*S*)-60 were found, and for 59f–j, which had acyl and sulfonyl *N*-protecting groups, (*R*)-configuration product (1*R*)-2,2,2-trichloroethyl 1-prop-2-enyl-1,9-dihydropyrido[3,4-*b*]indole-2-carboxylate (1*R*)-60 were noticed. Among 59a–e, the bulkier the *N*-protecting groups, the more ee was seen in (1*S*)-60 (*e.g.*, highest 91% ee in (*S*)-60 for 59e having anthracene-9-ylmethyl substituent; lowest 7% ee in (1*S*)-60 for 59a having methyl substituent). Among 59f–j, the bulkiness of substituents did not affect ee of (1*R*)-60 that much, only lowered the % yields (Scheme 16).

After that, silyl enol ethers 61a–e^76^ were used as nucleophiles instead of allyltributyltin. At 0 °C, reaction with (5*S*)-1-(anthracene-9-ylmethyl)-5-(pyrido[3,4-*b*]indole-9-carbonyl)pyrrolidin-2-one 58e and 61a reached only 40% yield with 79% ee of 2,2,2-trichloroethyl (1*S*)-1-(2-oxopropyl)-1,9-dihydropyrido[3,4-*b*]indole-2-carboxylate (1*S*)-62a in 24 hours; 61b needed 2.5 hours to reach 79% yield with 86% ee of 2,2,2-trichloroethyl (1*S*)-1-phenacyl-1,9-dihydropyrido[3,4-*b*]indole-2-carboxylate (1*S*)-62b; 61c gained quantitative yield only at 30 minutes with 82% ee of 2,2,2-trichloroethyl (1*S*)-1-(1-methoxy-2-methyl-1-oxopropan-2-yl)-1,9-dihydropyrido[3,4-*b*]indole-2-carboxylate (1*S*)-62c. Reducing the temperature to −40 °C reduced yields to 81 and 83% with 61d and 61e respectively even at reaction times of 12 and 19 hours but, increased ee slightly to 88 and 87% of 2,2,2-trichloroethyl (1*S*)-1-(2-oxo-2-phenylmethoxyethyl)-1,9-dihydropyrido[3,4-*b*]indole-2-carboxylate (1*S*)-62d and 2,2,2-trichloroethyl (1*S*)-1-(2-benzylsulfanyl-2-oxoethyl)-1,9-dihydropyrido[3,4-*b*]indole-2-carboxylate (1*S*)-62e respectively.

**Scheme 17:**
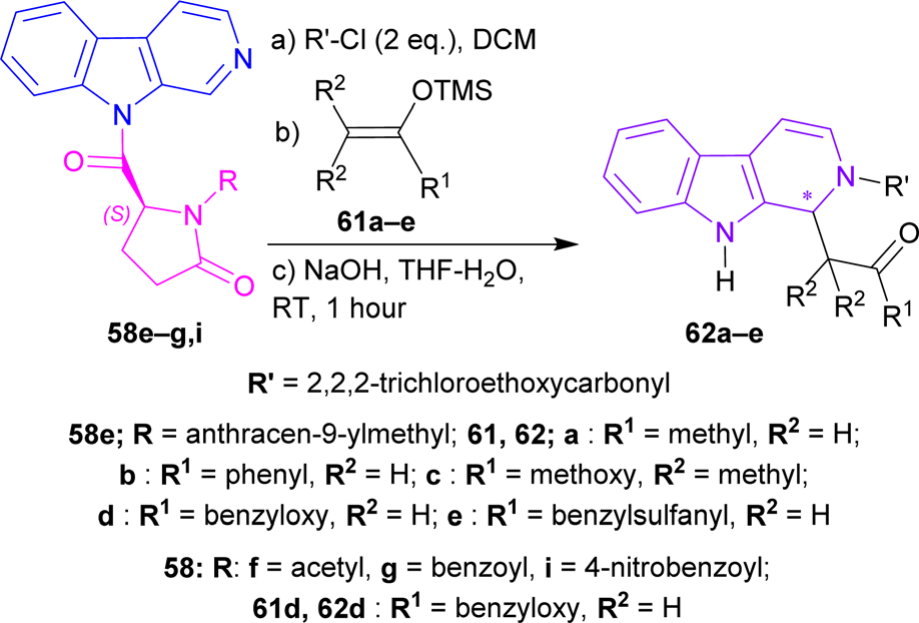
N2 protecting, C1 addition of silyl enol ether, and then ultimately removal of the chiral auxiliary.

61d was chosen to react with 58f,g,i at −78 °C for 40 hours to produce 2,2,2-trichloroethyl (1*R*)-1-(2-oxo-2-phenylmethoxyethyl)-1,9-dihydropyrido[3,4-*b*]indole-2-carboxylate (1*R*)-62d (75–76% ee). Less steric hindered acetyl-substitution 58f had a 93% yield of (1*R*)-62d but more steric hindered benzoyl-substitution 58g and 4-nitrobenzoyl-substitution 58i both had quantitative yields of (1*R*)-62d (Scheme 17).

**Scheme 18:**
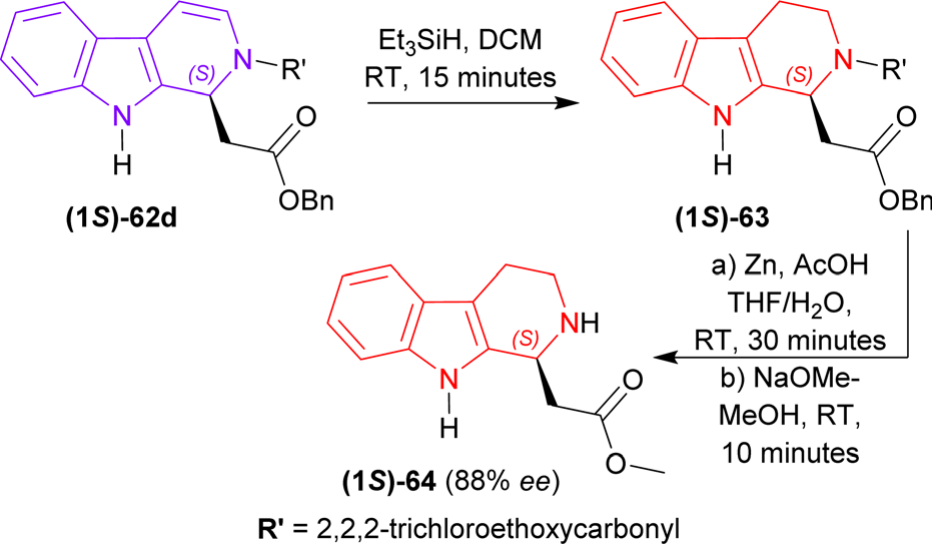
Reducing to THβC and removing N9 protection.

2,2,2-Trichloroethyl (1*S*)-1-(2-oxo-2-phenylmethoxyethyl)-THβC-2-carboxylate (1*S*)-62d was reduced with Et_3_SiH in DCM at RT for 15 minutes gave rise to (1*S*)-63 which was again reduced and *N*-2-deprotected with Zn-acetic acid (AcOH) to produce 92% yield of methyl 2-[(1*S*)-2,3,4,9-tetrahydro-1*H*-pyrido[3,4-*b*]indol-1-yl]acetate (1*S*)-64 (88% ee calculated according to Tietze *et al.*^77^) (Scheme 18).

So, (5*S*)-1-substituted-5-(β-carboline-9-carbonyl)pyrrolidin-2-one having alkyl N9-protecting groups as the chiral auxiliary ultimately produced (1*S*)-1-substituted-THβC.

#### Example 4. (1*R*)-1-Aryl-ethanamines as chiral auxiliaries

Siwicka *et al.* used (1*R*)-1-aryl-ethanamines as chiral auxiliaries to produce 1-substituted-THβCs.^78^

Tryptamine 13a with diethyl oxalate produced 65. (1*R*)-1-Phenylethanamine (1*R*)-66a^79^ and (1*R*)-1-naphthalen-1-ylethanamine (1*R*)-66b^80^ with 65, produced (*R*)-67a and (*R*)-67b. Bischler–Napieralski cyclization of (*R*)-67a,b with POCl_3_ in refluxing DCM gave (*R*)-68a and (*R*)-68b.

**Scheme 19:**
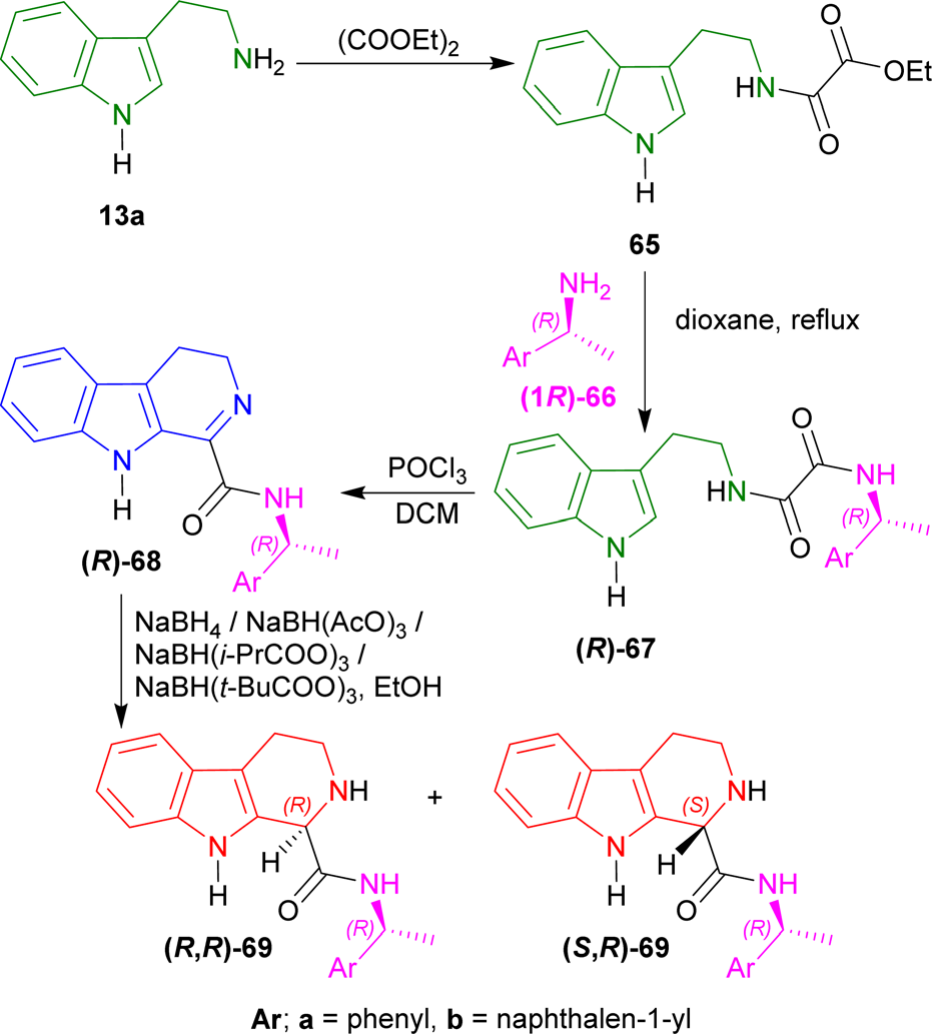
Addition of chiral auxiliary, Bischler–Napieralski cyclization, and reduction to THβC.

After that, several reducing agents were experimented with *e.g.*, sodium borohydride (NaBH_4_), sodium triacetoxy-borohydride (NaBH(AcO)_3_), sodium tris(2-methylpropanoyloxy)borohydride (NaBH(*i*-PrCOO)_3_), sodium tris(2,2-dimethylpropanoyloxy)borohydride (NaBH(*t*-BuCOO)_3_) in ethanol to produce dr of 62 : 38–78 : 22 for (1*R*)-*N*-[(1*R*)-1-phenylethyl]-THβC-1-carboxamide (*R*,*R*)-69a and (*S*,*R*)-69a from (*R*)-68a; and dr of 64 : 36–83 : 17 for (1*R*)-*N*-[(1*R*)-1-naphthalen-1-ylethyl]-THβC-1-carboxamide (*R*,*R*)-69b and (*S*,*R*)-69b from (*R*)-68b (Scheme 19).

So, the (*R*)-configured chiral auxiliary (1*R*)-1-phenylethanamine and (1*R*)-1-naphthalen-1-ylethanamine predominantly produced (1*R*)-1-substituted-THβCs.

#### Example 5. Using Ellman's sulfinamide as a chiral auxiliary to synthesize (−)-tetrahydroharman, (−)-komaroidine, (+)-*N*-methyltetrahydroharman, (+)-*N*-acetylkomaroidine, and (−)-harmicine

Reddy *et al.* used Ellman's sulfinamide as a chiral auxiliary to synthesize various chiral 1-substituted-THβCs.^81^

2-(1-Benzylindol-3-yl)ethanol 70a and 2-(1-methylindol-3-yl)ethanol 70b was reacted with POCl_3_ in DMF at 60 °C for 12 hours to produce 71a and 71b. Then 71a,b was refluxed with Ellman's sulfinamide or (*R*)-2-methylpropane-2-sulfinamide as chiral auxiliary,^82–85^ and Ti(OEt)_4_ in DCM for 24 hours to have 78% yield of (*R*,*E*)-72a and 79% yield of (*R*,*E*)-72b.^86^

**Scheme 20:**
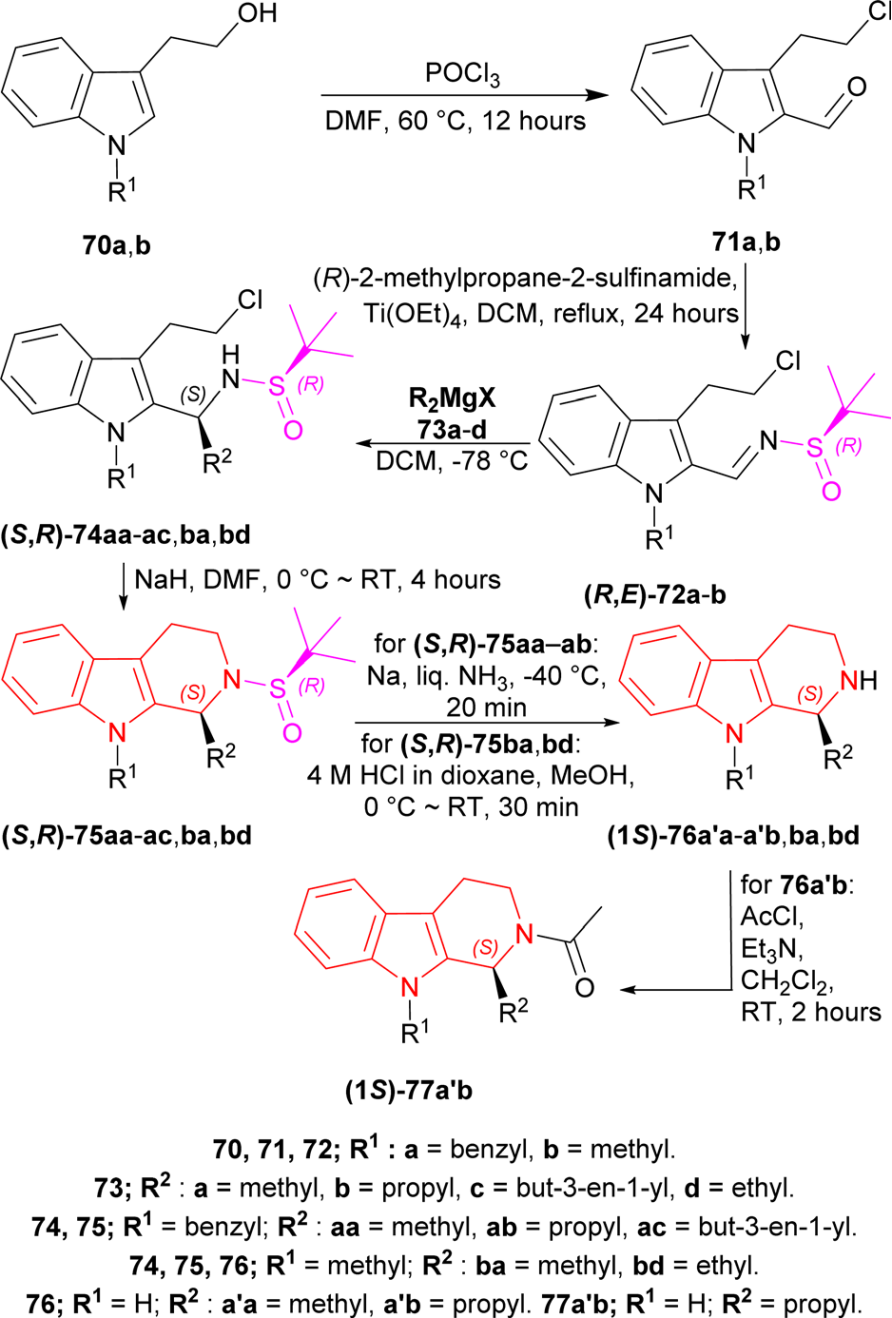
Addition of chiral auxiliary, base-catalyzed cyclization to THβC and removal of the chiral auxiliary.

**Scheme 21:**
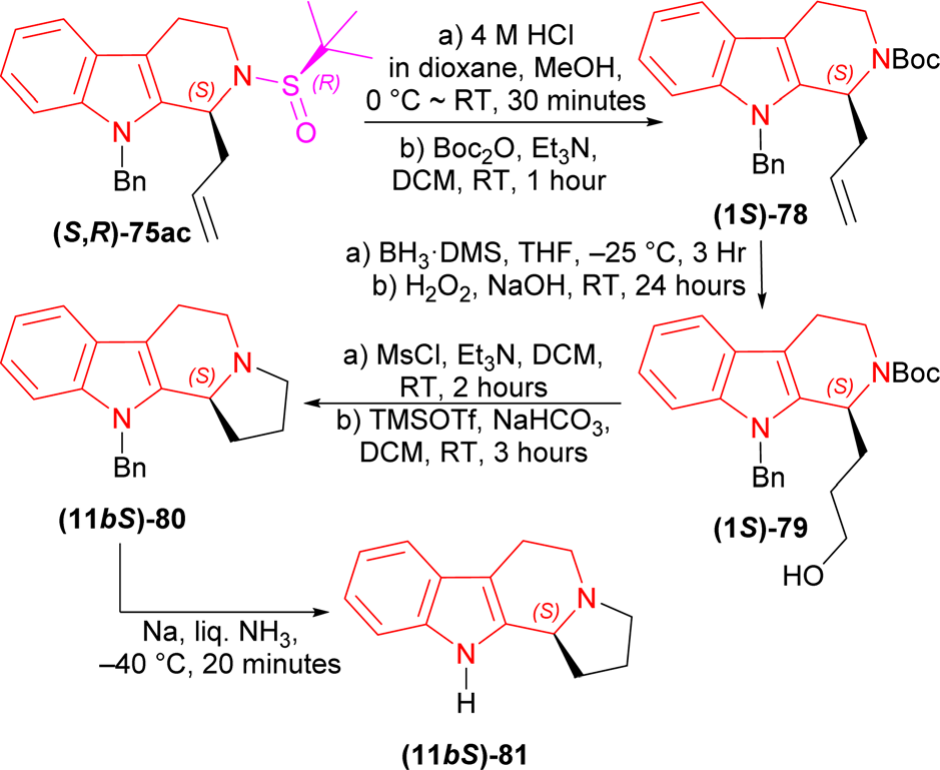
Removal of chiral auxiliary and protecting *N*-2, cyclization of the fourth ring, removal of *N*-9 protection.

Then it was experimented with various Grignard reagents 73a–d*e.g.*, MeMgI, PrMgBr, allyl magnesium bromide, and EtMgCl in DCM at −78 °C to have 77–84% yield and 84 to >98% de of (*S*,*R*)-74aa, (*S*,*R*)-74ab, (*S*,*R*)-74ac, (*S*,*R*)-74ba, (*S*,*R*)-74bd; among which (*S*,*R*)-74ac had the highest de of >98%.^87^ Base-catalyzed cyclization^88^ of (*S*,*R*)-74aa–ac,ba,bd with NaH in DMF at 0 °C to RT for 4 hours gave rise to 72–85% yield of (*S*,*R*)-75aa, (*S*,*R*)-75ab, (*S*,*R*)-75ac, (*S*,*R*)-75ba, (*S*,*R*)-75bd; among which (*S*,*R*)-75ac had the highest yield of 85%.

(*S*,*R*)-75aa,ab with Na in liquid NH_3_ (ref. 89) at −40 °C for 20 minutes removed the chiral auxiliary, N9-benzyl and produced 70% yield of (−)-tetrahydroharman (1*S*)-76a′a and 68% yield of (−)-komaroidine (1*S*)-76a′b. (*S*,*R*)-75ba,bd with 4 M HCl in dioxane in MeOH at 0 °C to RT for 30 minutes removed the chiral auxiliary and produced 87% yield of (+)-*N*-methyltetrahydroharman (1*S*)-76ba and 83% yield of (1*S*)-1-ethyl-9-methyl-1,2,3,4-tetrahydropyrido[3,4-*b*]indole (1*S*)-76bd.

(1*S*)-76a′b with acetyl chloride and Et_3_N in DCM at RT for 2 hours produced an 87% yield of (+)-*N*-acetylkomaroidine (1*S*)-77a′b (Scheme 20).

(1*S*)-9-Benzyl-2-[(*R*)-*tert*-butylsulfinyl]-1-prop-2-enyl-THβC (*S*,*R*)-75ac with 4 M HCl in dioxane in MeOH solvent at 0 °C to RT for 30 minutes; *tert*-butyl (2-methylpropan-2-yl)oxycarbonyl carbonate (Boc_2_O) and Et_3_N in DCM at RT for 1 hour produced 88% yield of (1*S*)-78. Then (1*S*)-78 with BH_3_·DMS in THF at −25 °C for 3 hours; H_2_O_2_ in NaOH at RT for 24 hours gave 83% yield of (1*S*)-79. After that, (1*S*)-79 with methanesulfonyl chloride (MsCl) and Et_3_N in DCM at RT for 2 hours; TMSOTf and NaHCO_3_ in DCM at RT for 3 hours had 70% yield of (11*bS*)-80. Lastly, (11*bS*)-80 with Na in liquid NH_3_ at −40 °C for 20 minutes gave the ultimate product (−)-harmicine (11*bS*)-81 of 72% yield (Scheme 21).

So, Ellman's sulfinamide as chiral auxiliary produced (1*S*)-1-substituted-THβCs *e.g.*, (−)-tetrahydroharman, (−)-komaroidine, (+)-*N*-methyltetrahydroharman, (+)-*N*-acetylkomaroidine, and (−)-harmicine which all have various important pharmacological activities.^12,90^

### Method 3. Asymmetric transfer hydrogenation reaction with chiral catalysts

The transfer hydrogenation reaction, which involves the addition of hydrogen to a molecule from a non-H_2_ source, is a versatile and effective approach for producing various hydrogenated compounds. This method is gaining popularity in hydrogenation research as an appealing alternative to direct hydrogenation. The key reasons for its growing interest include: (i) it eliminates the need for potentially hazardous pressurized H_2_ gas and complex experimental setups, (ii) the hydrogen donors used are typically affordable, easy to handle, and widely available, (iii) the main byproduct can often be recycled, and (iv) the catalysts involved are generally easy to obtain and not highly sensitive.^91–101^

ATH emerged in the early 1980s. The first reports were of the Ru catalyzed ATH.^93,102,103^ ATH that used the late transition-metal catalysts has shown to be one of the most potent strategies for asymmetric reduction of diverse unsaturated substrates to create chiral chemicals.^94,95,97,98,104–107^

#### Example 1. ATH to synthesize 1-alkyl-1,2,3,4-tetrahydropyrido[3,4-*b*]indole

**Fig. 3:**
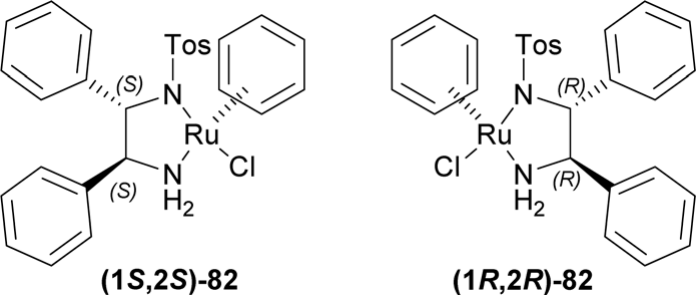
Chiral catalysts for ATH.

Roszkowski *et al.* used (1*S*,2*S*)-82 and (1*R*,2*R*)-82 as chiral catalysts for the ATH of 1-alkyl-3,4-dihydropyrido[3,4-*b*]indole (Fig. 3).^108^

**Scheme 22:**
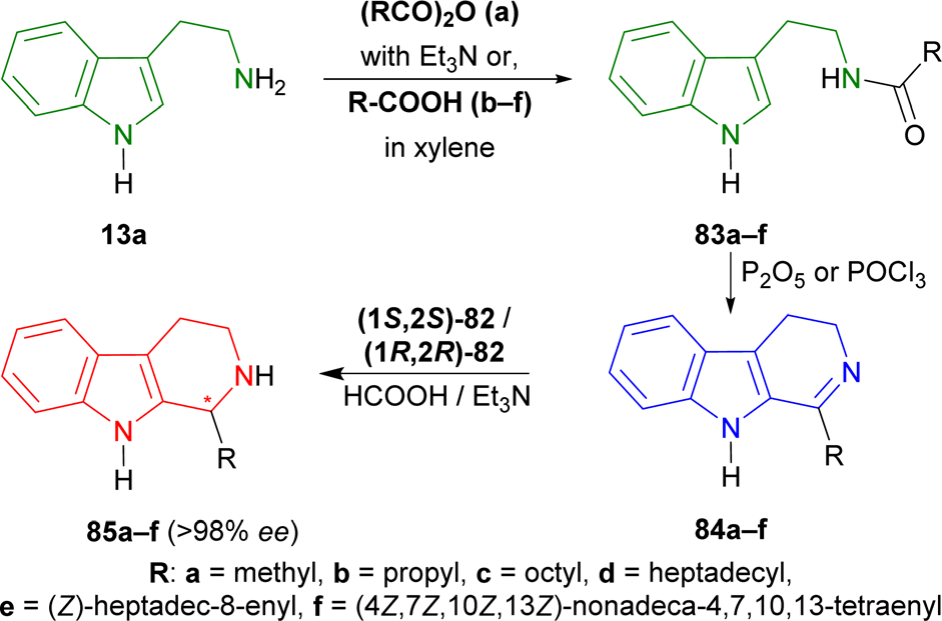
Synthesizing *N*-[2-(1*H*-indol-3-yl)ethyl]amides from tryptamine, then 1-substituted-DHβCs, and ultimately ATH to get 1-substituted-THβCs with chiral catalysts.

Tryptamine 13a was reacted with acetic anhydride with Et_3_N, butyric acid in xylene, nonanoic acid in xylene, stearic acid in xylene, oleic acid, and arachidonic acid to produce the 83a–f.^109^ With P_2_O_5_ or POCl_3_, Bischler–Napieralski cyclization produces 84a–f which instantly underwent ATH^34^ with (1*S*,2*S*)-82 and (1*R*,2*R*)-82.^77^ All ATH products had >98% ee. For 84a–f, (1*S*,2*S*)-82 gave 70–85% yields of (1*R*)-1-substituted-THβCs (1*R*)-85a–f; and for 84a–e, (1R,2*R*)-82 gave 77–88% yields of (1*S*)-1-substituted-THβCs (1*S*)-85a–e. Highest yield of 88% was found for (1*S*)-1-propyl-THβC (1*S*)-85b and lowest yield of 70% for (1*R*)-1-[(4*Z*,7*Z*,10*Z*,13*Z*)-nonadeca-4,7,10,13-tetraenyl]-THβC (1*R*)-85f having highly sterically hindered substituents. Switching catalyst from (1*S*,2*S*)-82 to (1R,2*R*)-82 lowered the % yields of the products of (1*S*)-85a,c,d slightly by 2–4% from that of (1*R*)-85a,c,d. (1*S*)-85b had 9% more yield than (1*R*)-1-propyl-THβC (1*R*)-85b while (1*S*)-1-[(*Z*)-heptadec-8-enyl]-THβC (1*S*)-85e had 7% less yield than (1*R*)-1-[(*Z*)-heptadec-8-enyl]-THβC (1*R*)-85e (Scheme 22).

So, (1*S*,2*S*)-82 chiral catalyst produced (1*R*)-1-substituted-THβCs and (1*R*,2*R*)-82 chiral catalyst produced (1*S*)-1-substituted-THβCs predominantly.

#### Example 2. ATH to synthesize of (*R*)-harmicine and (*R*)-desbromoarborescidine A

Szawkało *et al.* used (1*S*,2*S*)-82 (Fig. 3) for the ATH to synthesize of (*R*)-harmicine and (*R*)-desbromoarborescidine A.^110^

**Scheme 23:**
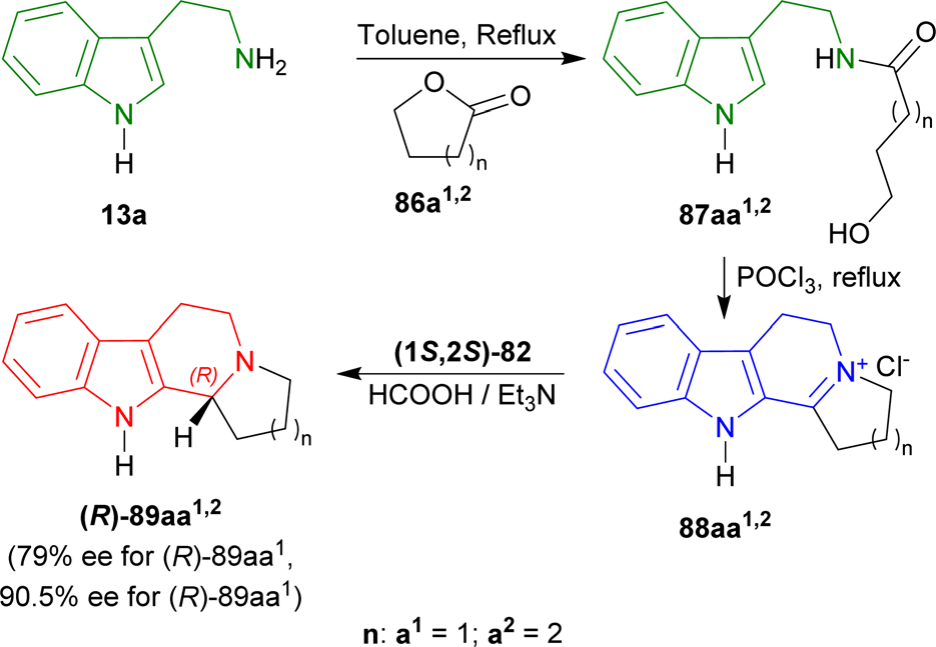
Synthesizing *N*-[2-(1*H*-indol-3-yl)ethyl]hydroxamides from tryptamine, then 1-substituted-DHβCs, and ultimately ATH to get 1-substituted-THβCs with chiral catalysts.

Oxolan-2-one (γ-butyrolactone) 86a^1^, and oxan-2-one (δ-valerolactone) 86a^2^ were treated with tryptamine 13a produced 87aa^1^ (ref. 111) (78% yield), and 87aa^2^ (ref. 112) (87% yield). Then Bischler–Napieralski cyclization in POCl_3_ gave iminium salts 88aa^1^ and 88aa^2^. Immediate ATH of 88aa^1,2^ with (1*S*,2*S*)-82 ultimately gave rise to (11*bR*)-2,3,5,6,11,11*b*-hexahydro-1*H*-indolizino[8,7-*b*]indole or (*R*)-harmicine (*R*)-89aa^1^ (81% yield, 79% ee) and (12*bR*)-1,2,3,4,6,7,12,12*b*-octahydroindolo[2,3-*a*]quinolizine or (*R*)-desbromoarborescidine A (*R*)-89aa^2^ (84% yield, 90.5% ee) (Scheme 23).

So, (1*S*,2*S*)-82 chiral catalyst produced (1*R*)-1-substituted-THβCs predominantly.

#### Example 3. ATH to synthesize of (*R*)-trypargine

Czarnocki *et al*. used (1*S*,2*S*)-82 (Fig. 3) to synthesize (*R*)-trypargine *via* ATH.^113^

**Scheme 24:**
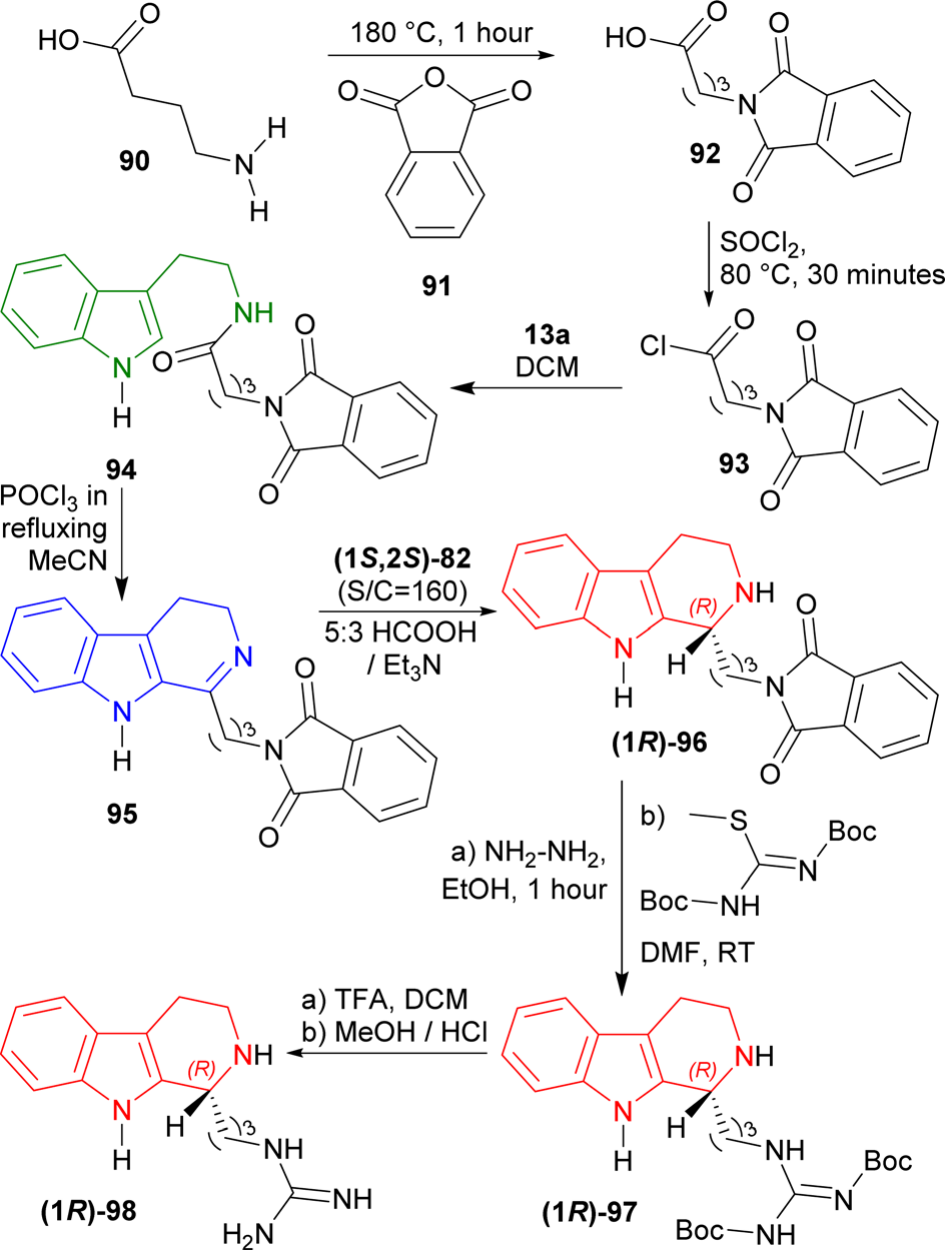
ATH with chiral catalysts to synthesize HCl salt of (*R*)-trypargine.

4-Aminobutanoic acid 90 was treated with 2-benzofuran-1,3-dione (phthalic anhydride) 91 at 180 °C for 1 hour^114^ to give 92 which was turned into 93 with sulfonyl chloride at 80 °C for 30 minutes. This was reacted with 13a in DCM to get 82% yield of 94. It was reacted with POCl_3_ in refluxing acetone (MeCN) to give 85% yield of 95*via* Bischler–Napieralski reaction. After that, ATH of 95 with (1*S*,2*S*)-82 (S : C ratio of 160 : 1) in 5 : 3 azeotropic solution of formic acid (HCOOH) : Et_3_N^34^ afforded 92% yield of (1*R*)-96 (>98% ee).

(1*R*)-96 was reacted with hydrazine in ethanol at RT for 1 hour to remove the phthaloyl group which was readily subjected to *tert*-butyl *N*-[[(2-methylpropan-2-yl)oxycarbonylamino]-methylsulfanylmethylidene]carbamate in DMF at RT to get 53% yield of (1*R*)-97. At the last step, Boc group of (1*R*)-97 was removed by TFA in DCM at RT, and successive evaporation with methanolic HCl provided the final product HCl salt of (*R*)-trypargine (1*R*)-98 in quantitative yield. The isolated compound's analytical results were entirely consistent with what was previously published by Cesar *et al.*^115^ (Scheme 24).

So, (1*S*,2*S*)-82 chiral catalyst produced HCl salt of (*R*)-trypargine predominantly.

#### Example 4. Asymmetric hydrogen-transfer to synthesize eudistomidin B and it's diastereomer

Takahashi *et al.* used (1*S*,2*S*)-82 and (1*R*,2*R*)-82 (Fig. 3) to synthesize eudistomidin B and it's diastereomer *via* asymmetric hydrogen-transfer.^116^

**Scheme 25:**
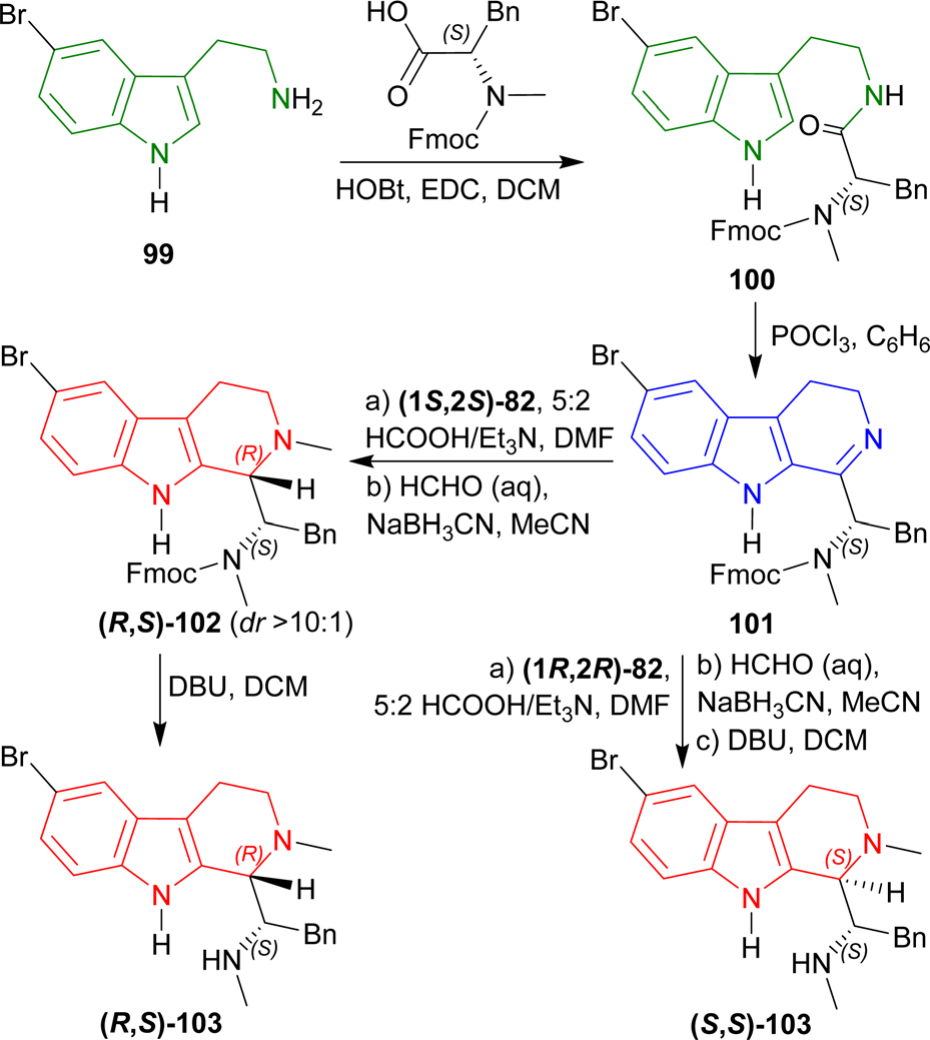
Asymmetric synthesis of eudistomidin B and it's diastereomer.

2-(5-Bromo-1*H*-indol-3-yl)ethanamine 99 (ref. 117) with (2*S*)-2-[9*H*-fluoren-9-ylmethoxycarbonyl(methyl)amino]-3-phenylpropanoic acid,^118^1,2-dichloroethane (EDC), and HOBt in DCM produced 100. Bischler–Napieralski cyclization^119^ of 100 in benzene gave rise to 101. The compound 101 was then treated with (1*S*,2*S*)-82 followed by 5 : 2 HCOOH/Et_3_N in DMF and *N*-2 was methylated with aq. HCHO, NaBH_3_CN, in CH_3_CN into (*R*,*S*)-102 with 89% yield (predominating (*S*,*S*)-102 by >10 : 1 dr). The Fmoc group was removed by 2,3,4,6,7,8,9,10-octahydropyrimido[1,2-*a*]azepine (DBU) in DCM to produce (1*S*)-1-[(1*R*)-6-bromo-2-methyl-1,3,4,9-tetrahydropyrido[3,4-*b*]indol-1-yl]-*N*-methyl-2-phenylethanamine (*R*,*S*)-103 (eudistomidin B) in 85% yield. But when (1*R*,2*R*)-82 was used, (1*S*)-1-[(1*S*)-6-bromo-2-methyl-1,3,4,9-tetrahydropyrido[3,4-*b*]indol-1-yl]-*N*-methyl-2-phenylethanamine (*S*,*S*)-103 (diastereomer of eudistomidin B) was found in 78% yield (Scheme 25).

So, (1*S*,2*S*)-82 chiral catalyst produced (1*R*)-1-substituted-THβC eudistomidin B; and (1*R*,2*R*)-82 chiral catalyst produced (1*S*)-1-substituted-THβC, the (*S*,*S*)-diastereomer of eudistomidin B.

#### Example 5. Transfer hydrogenation reaction of hydroxylactams catalyzed by chiral phosphoric acid

**Fig. 4:**
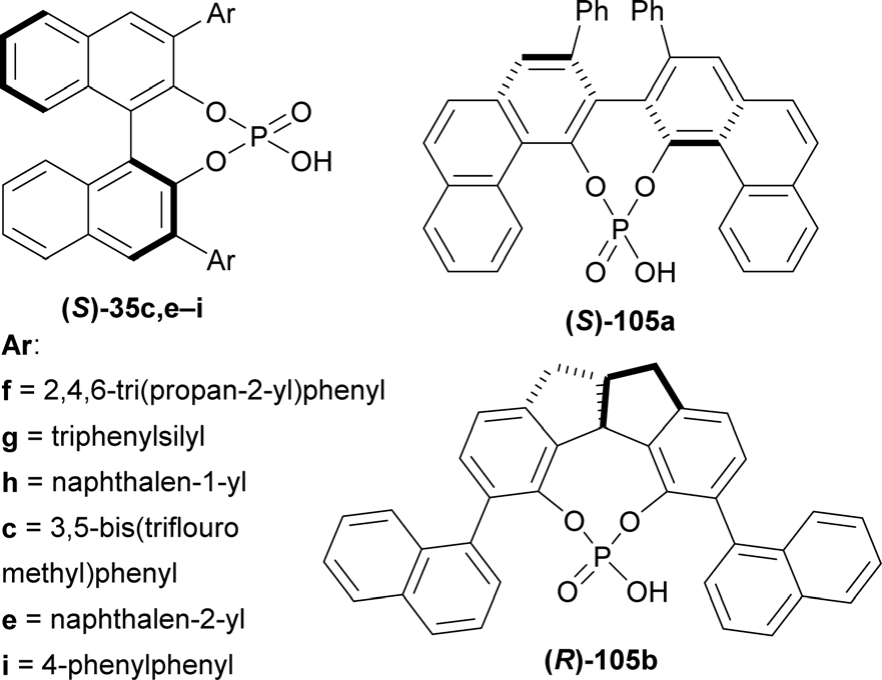
(*S*)-BINOL, VAPOL, and SPINOL derived chiral phosphoric acid.

Yin *et al.* used (*S*)-BINOL, VAPOL, and SPINOL-derived chiral phosphoric acid catalysts (Fig. 4) for ATH of hydroxylactams.^120^

**Scheme 26:**
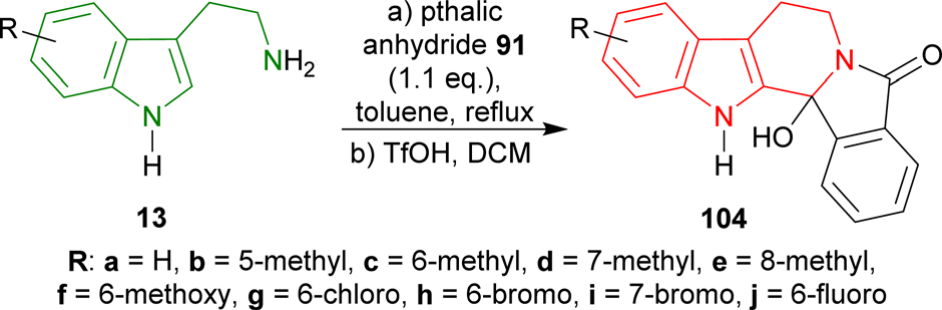
Synthesis of hydroxylactams from tryptamine and its derivatives.

**Scheme 27:**
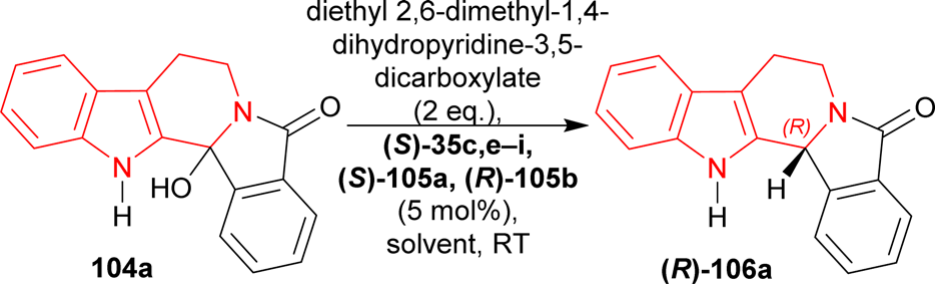
Screening of chiral phosphoric acid catalysts for the acid catalyzed ATH of hydroxylactam.

Tryptamine and its derivatives 13a–j were refluxed with phthalic anhydride 91 in toluene, and trifluoromethanesulfonic acid (TfOH) in DCM to give 2-hydroxy-10,20-diazapentacyclo[11.7.0.0^2,10^.0^3,8^.0^14,19^]icosa-1(13),3,5,7,14,16,18-heptaen-9-one and its derivatives 104a–j in 39–63% yields (Scheme 26).^121,122^

(*S*)-BINOL, VAPOL, and SPINOL-derived chiral phosphoric acid catalysts (*S*)-35c,e–i, (*S*)-105a, and (*R*)-105b^123,124^ (Fig. 4) respectively were tested for transfer hydrogenation reaction of 2-hydroxy-10,20-diazapentacyclo[11.7.0.0^2,10^.0^3,8^.0^14,19^]icosa-1(13),3,5,7,14,16,18-heptaen-9-one 104a with a Hantzsch ester (diethyl 2,6-dimethyl-1,4-dihydropyridine-3,5-dicarboxylate, 2 eq.) as the hydride source to give (2*R*)-10,20-diazapentacyclo[11.7.0.0^2,10^.0^3,8^.0^14,19^]icosa-1(13),3,5,7,14,16,18-heptaen-9-one (*R*)-106a in DCM at RT. Among the catalysts, (*S*)-35g provided with highest ee of 52% with 88% yield of the product. After that, different solvents were tested among which dioxane had highest ee of 75% with 84% yield (Scheme 27).

**Scheme 28:**
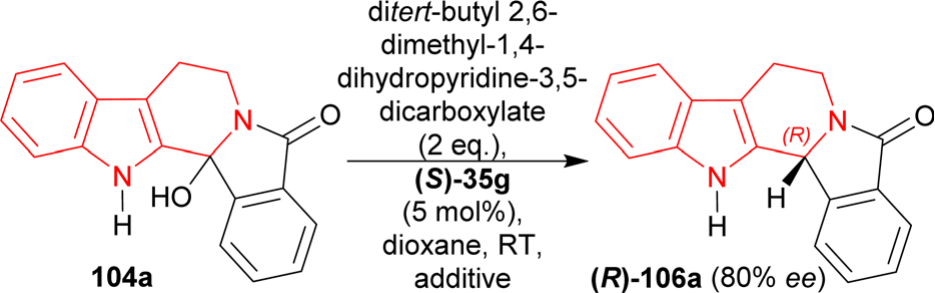
Screening of additive for the acid catalyzed ATH of hydroxylactam.

Then, a different Hantzsch ester (di*tert*-butyl 2,6-dimethyl-1,4-dihydropyridine-3,5-dicarboxylate, 2 eq.) was tested as hydride donor with the presence of (*S*)-35g that resulted in 91% yield and 80% ee of (*R*)-106a from 104a in 24 hours. But reactions using 3, 4, or 5 Å meshes and magnesium sulphate did not increase the yield or ee (Scheme 28).

**Scheme 29:**
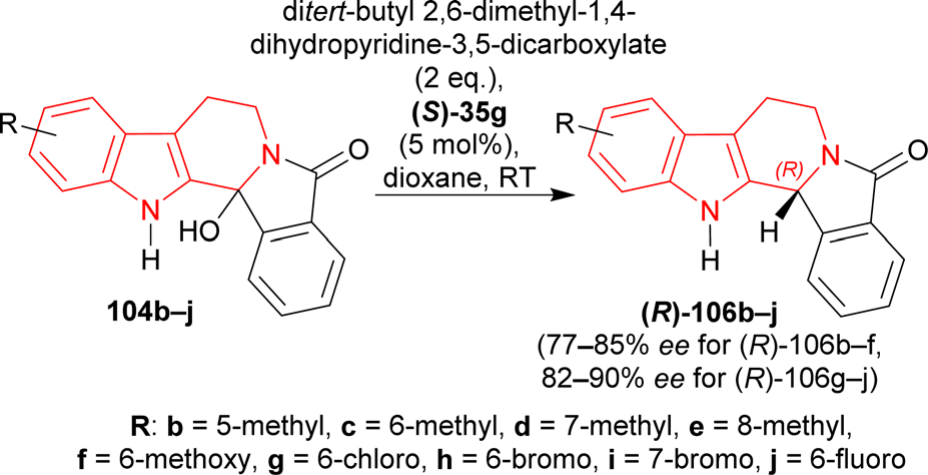
Acid catalyzed ATH reaction of hydroxylactams by (*S*)-35g under the optimized conditions.

Under the optimized conditions, 104b–j were converted to (*R*)-106b–j with (*S*)-35g; among which (*R*)-106b–f containing an electron-rich group had 68–93% yields with 77–85% ee, and (*R*)-106g–j containing an electron-poor group had 90–94% yields with 82–90% ee (Scheme 29).

So, (*S*)-BINOL-derived chiral phosphoric acid produced (1*R*)-1-substituted-THβCs predominantly.

#### Example 6. Total synthesis of (+)-reserpine by primary amine catalysts and [Ir(COD)(PCy_3_)(py)]BAr_F_

Rajapaksa *et al.* synthesized (+)-reserpine with the help of primary amine catalysts and [Ir(COD)(PCy_3_)(py)]BAr_F_ as a chiral catalyst for ATH.^42^

**Fig. 5:**
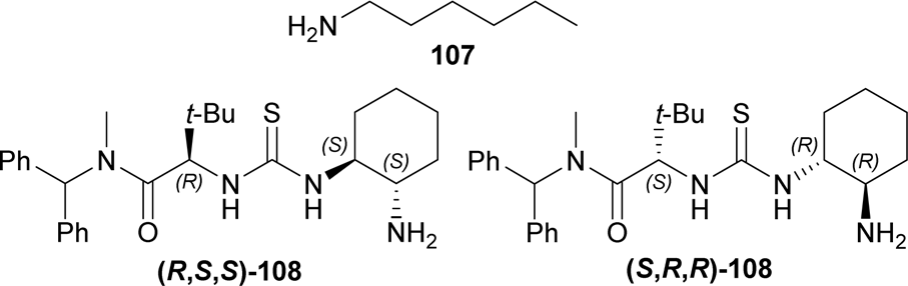
1° amine catalysts for coupling of 7-methoxy-9-tosyl-DHβC.

**Scheme 30:**
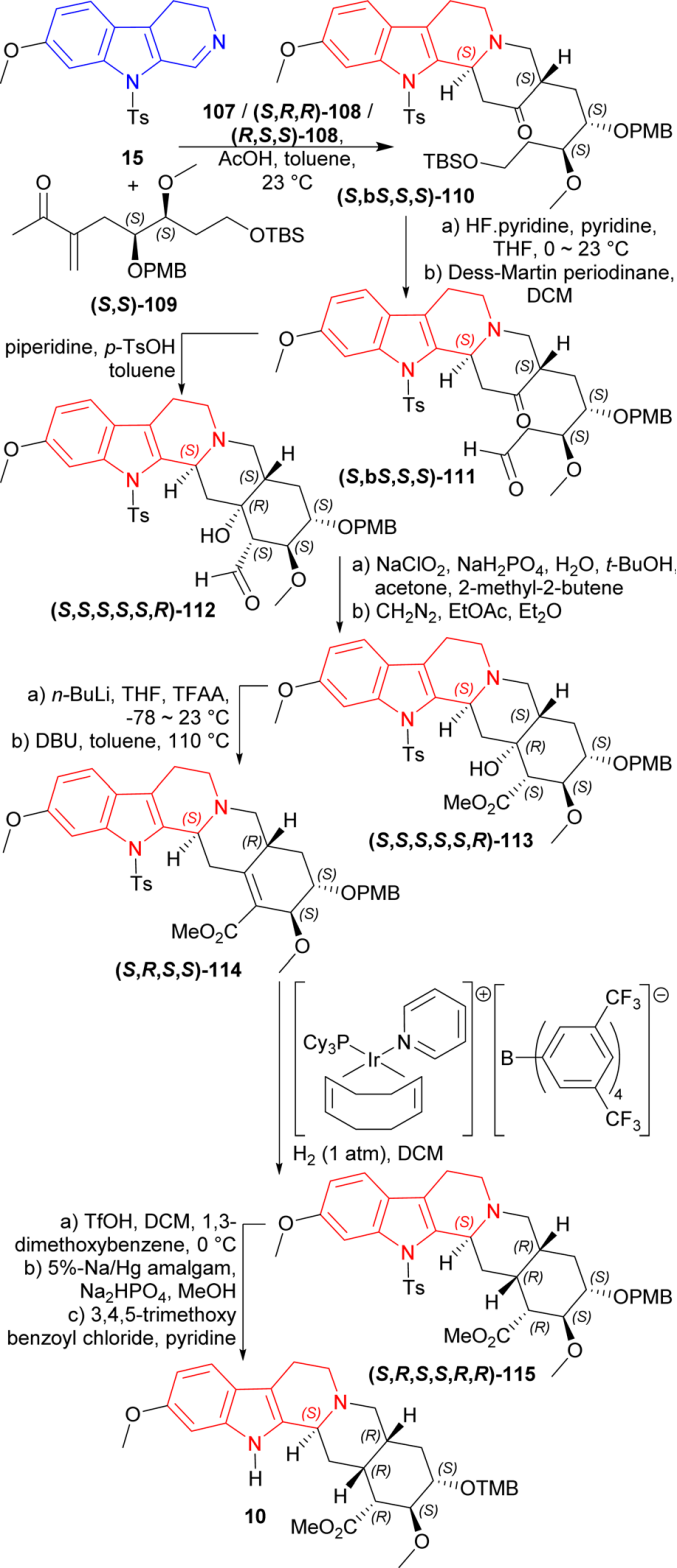
Total synthesis of (+)-reserpine by primary amine catalysts and [Ir(COD)(PCy_3_)(py)]BAr_F_.

1° amine catalyst *e.g.*, hexan-1-amine 107, (2*S*)-2-[[(1*R*,2*R*)-2-aminocyclohexyl]carbamothioylamino]-*N*-benzhydryl-*N*,3,3-trimethylbutanamide (*S*,*R*,*R*)-108 or (2*R*)-2-[[(1*S*,2*S*)-2-aminocyclohexyl]carbamothioylamino]-*N*-benzhydryl-*N*,3,3-trimethylbutanamide (*R*,*S*,*S*)-108 (Fig. 5) were used to couple 7-methoxy-9-(4-methylphenyl)sulfonyl-3,4-dihydropyrido[3,4-*b*]indole (7-methoxy-9-tosyl-DHβC) 15 with (*S*,*S*)-109 (ref. 125) (1.2 eq.)^126,127^ around acetic acid in toluene at 23 °C. 100 mol% of 107 had 90% conversion rate after 9 Days with 1 : 1 dr of the (*S*,*bS*,*S*,*S*)-110 and (*R*,*bR*,*S*,*S*)-110. 20 mol% of (*S*,*R*,*R*)-108 (ref. 125) produced >99% conversion after 6 days with 11.5 : 1 : 1.8 dr of the (*S*,*bS*,*S*,*S*)-110, (*R*,*bR*,*S*,*S*)-110, and (*R*,*bS*,*S*,*S*)-110; ultimately yielding 76% of the desired (*S*,*bS*,*S*,*S*)-110. Here, the use of 20 mol% (*R*,*S*,*S*)-108 produced greater dr for (*R*,*bR*,*S*,*S*)-110. The cleavage of the primary TBS ether of (*S*,*bS*,*S*,*S*)-110 was done in two steps by pyridine-buffered HF in pyridine at 0 °C to 23 °C; and then oxidation with the Dess–Martin periodinane in DCM producing (*S*,*bS*,*S*,*S*)-111. Piperidine and *p*-TsOH was treated with (*S*,b*S*,*S*,*S*)-111 to produce an intramolecular enamine aldol (*S*,*S*,*S*,*S*,*S*,*R*)-112. Pinnick oxidation followed by esterification with diazomethane of (*S*,*S*,*S*,*S*,*S*,*R*)-112 provided (*S*,*S*,*S*,*S*,*S*,*R*)-113. Trifluoroacetylation of (*S*,*S*,*S*,*S*,*S*,*R*)-113 and subsequent elimination by DBU gave (*S*,*R*,*S*,*S*)-114. Hydrogenation with H_2_ (1 atm) in DCM and [Ir(COD)(PCy_3_)(py)]BAr_F_ (ref. 128 and 129) gave 6 : 1 dr of (*S*,*R*,*S*,*S*,*R*,*R*)-115 (44% isolated yield) and (*S*,*R*,*S*,*S*,*R*,*S*)-115. Treating (*S*,*R*,*S*,*S*,*R*,*R*)-115 with TfOH, sodium–mercury amalgam, and 3,4,5-trimethoxy benzoyl chloride^130^ resulted in cleavage of PMB ether (86% yield), cleavage of tosyl protective group (69% yield), and esterification (90% yield) respectively; which ultimately gave (+)-reserpine 10 (Scheme 30).

So, 1° amine catalysts and [Ir(COD)(PCy_3_)(py)]BAr_F_ created (1*S*) and (19*R*,20*R*)-chiral centers predominantly to produce (+)-reserpine ultimately.

### Method 4. Asymmetric addition reaction

(*S*)-Proline catalyzed asymmetric addition reactions^131,132^ are getting popularized day-by-day. In this method, unsaturated C1 and N2 get saturated and 1-substituted-THβCs are produced with the help of a ketone. Besides that, cycloaddition^133^ is also being used highly in synthetic organic chemistry,^134,135^ specially [3 + 3] cycloaddition to produce heterocyclic compounds.^136–140^

#### Example 1. Asymmetric addition reaction of 9-tosyl-DHβC to synthesize the precursor of yohimbine and deserpidine catalyzed by (*S*)-proline

Itoh *et al.* synthesized precursor of yohimbine and deserpidine by asymmetric addition reaction of 9-tosyl-DHβC with ketones and proline-catalyzation.^43^

9-Tosyl-DHβC 16 (ref. 141) was reacted with 20% (v/v) of MeCN, (*S*)-proline (30 mol%) in DCM, MeCN, or DMSO at RT for 1.5–2 hours to produce 1-[(1*R*)-9-(4-methylphenyl)sulfonyl-1,2,3,4-tetrahydropyrido[3,4-*b*]indol-1-yl]propan-2-one (1*R*)-116a in good to quantitative yield but ee was very low (5–34%). After adding 10 eq. of water in each solvent, ee increased significantly (67–80%) but reaction time also increased (2–3.5 hours). For DCM, increasing water to 50 eq. did not help at all (trace yield). For MeCN, increasing water to 50 eq. increased ee only 2% (with quantitative yield), though 100 eq. of water decreased both the yield and ee. Lastly for DMSO, increasing water gradually to 50, 100, and 150 eq. increased ee to 80, 86, and 87%. So, DMSO was chosen as the solvent.

**Scheme 31:**
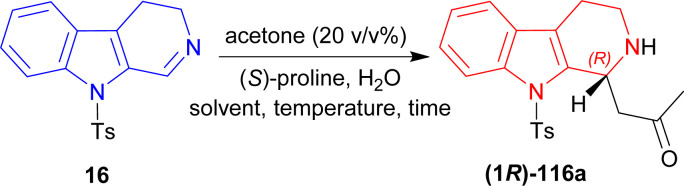
Screening of solvent, temperature, and time.

Then at −2 °C, the lowest temperature at which the solvent remained liquid, 50 and 100 eq. of water produced similar ee (92–93%) with increasing yields (91 and 99% respectively). Decreasing (*S*)-Proline to 3 mol% did not decrease the yield, but increasing water from 2 to 10 eq. at RT increased ee from 4 to 60%; and increasing water to 50 eq. at −2 °C required 23 hours to get 99% yield with 94% ee (Scheme 31).

Then butan-2-one, pentan-2-one, and 4-(2-methylpropoxy)butan-2-one was used instead of MeCN with 50 mol% of (*S*)-proline. Without water, the products had low ee of 7–28% with 57–78% yields at RT for reaction times of 4–26 hours. With 10 eq. of water, 1-[(1*R*)-9-(4-methylphenyl)sulfonyl-1,2,3,4-tetrahydropyrido[3,4-*b*]indol-1-yl]butan-2-one (1*R*)-116b and 1-[(1*R*)-9-(4-methylphenyl)sulfonyl-1,2,3,4-tetrahydropyrido[3,4-*b*]indol-1-yl]-4-(2-methylpropoxy)butan-2-one (1*R*)-116d had 51–80% ee with 65–81% yields at RT after 8–20 hours of reaction. For 50 eq. of water, products had 75–88% ee with 51–81% yields at RT after 7–20 hours reaction times; at −2 °C, (1*R*)-116b and 1-[(1*R*)-9-(4-methylphenyl)sulfonyl-1,2,3,4-tetrahydropyrido[3,4-*b*]indol-1-yl]pentan-2-one (1*R*)-116c had 89–92% ee with 76–85% yields after reacting 30–48 hours.

**Scheme 32:**
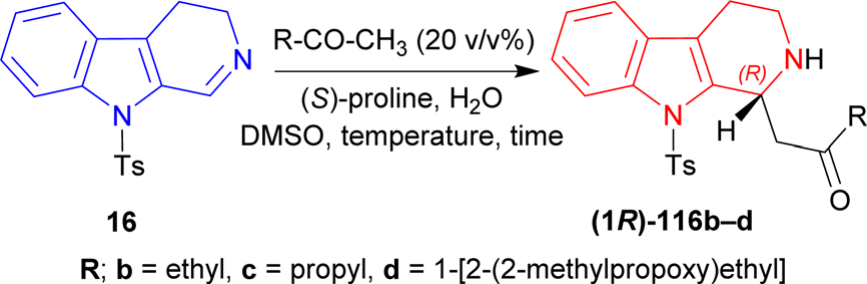
Screening of ketones.

When 5 mol% of (*S*)-proline was used with 50 eq. of water at RT, (1*R*)-116b and (1*R*)-116c required 36–72 hours to reach 85% ee with 86–98% yield; at −2 °C, (1*R*)-116b and (1*R*)-116c required 120 hours to reach 66–81% ee with 91–92% yield (Scheme 32).

**Scheme 33:**
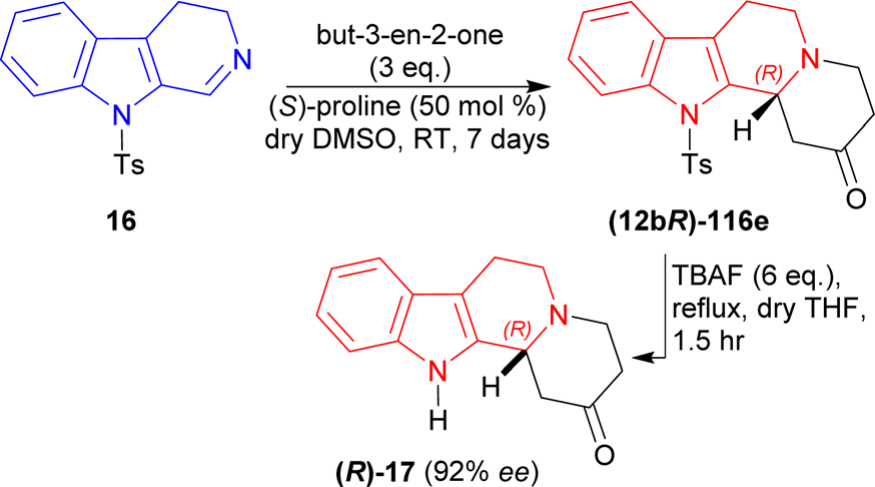
Synthesis of the precursor of yohimbine and deserpidine.

Then, 3 eq. of but-3-en-2-one was reacted with 16 in dry DMSO with (*S*)-proline at RT for 7 days to give (12*bR*)-116e (76% yield, 92% ee). Then (12*bR*)-116e was refluxed with 6 eq. of tetrabutylazanium;fluoride (TBAF) in dry THF for 1.5 hours to yield 74% (12*bR*)-3,4,6,7,12,12*b*-hexahydro-1*H*-indolo[2,3-*a*]quinolizin-2-one (*R*)-17 (ref. 142) (92% ee) which has been used as a precursor for the synthesis of yohimbine^143^ and deserpidine^144^ (Scheme 33).

So, but-3-en-2-one was asymmetrically added to 9-tosyl-DHβC by (*S*)-proline catalysis to synthesize a (1*R*)-1-substituted-THβC, the precursor of yohimbine and deserpidine.

#### Example 2. Synthesis of enantiomer of dihydrocorynantheol

Itoh *et al.* synthesized enantiomer of dihydrocorynantheol by asymmetric addition reaction of 9-tosyl-DHβC with ketones and proline-catalyzation.^126^

**Scheme 34:**
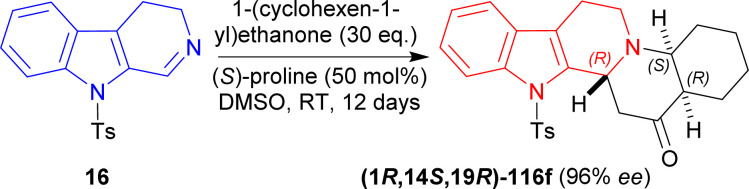
Generating three chiral centers in a single step (*S*)-proline catalyzed asymmetric addition reaction.

16 was reacted with 30 eq. of 1-(cyclohexen-1-yl)ethenone, 50 mol% of (*S*)-proline in DMSO at RT for 12 day to produce 91% yield and 96% ee of (1*R*,14*S*,19*R*)-3-(4-methylphenyl)sulfonyl-3,13-diazapentacyclo[11.8.0.0^2,10^.0^4,9^.0^14,19^]henicosa-2(10),4,6,8-tetraen-20-one (1*R*,14*S*,19*R*)-116f (Scheme 34).

**Scheme 35:**
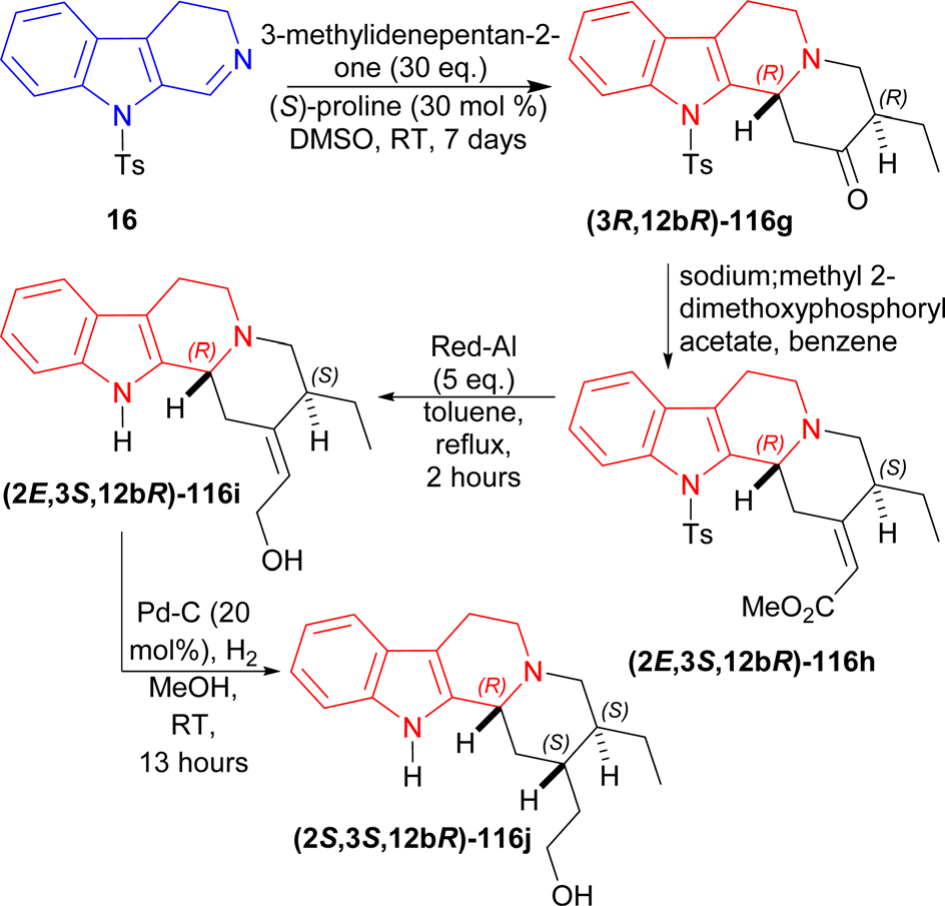
Synthesizing enantiomer of dihydrocorynantheol by (*S*)-proline catalyzed asymmetric addition reaction in four steps.

16 was reacted with 30 eq. of 3-methylidenepentan-2-one,^145^ 50 mol% of (*S*)-proline in DMSO at RT for 7 days to produce (3*R*,12*bR*)-116g (85% yield, 99% ee). After that, (3*R*,12*bR*)-116g was reacted with a Wittig reagent (sodium;methyl 2-dimethoxyphosphorylacetate) in benzene to have (2*E*,3*S*,12*bR*)-116h in very much higher ratio than its *Z* isomer (*E* : *Z* = 20 : 1). Then reflux with 5 eq. of Red-Al in DCM for 2 hours reduced (2*E*,3*R*,12*bR*)-116h to (2*E*,3*S*,12*bR*)-116i (63% yield). Lastly hydrogenation of (2*E*,3*S*,12*bR*)-116i with H_2_ in presence of 20 mol% Pd–C in methanol at RT for 13 hours yielded 74% (38% total yield) of 2-[(2*S*,3*S*,12*bR*)-3-ethyl-1,2,3,4,6,7,12,12*b*-octahydroindolo[2,3-*a*]quinolizin-2-yl]ethanol (2*S*,3*S*,12*bR*)-116j (enantiomer of dihydrocorynantheol) (Scheme 35).

So, 1-(cyclohexen-1-yl)ethenone and 3-methylidenepentan-2-one were asymmetrically added to 9-tosyl-DHβC by (*S*)-proline catalysis to synthesize (1*R*)-1-substituted-THβCs in one step and four steps respectively.

#### Example 3. Catalytic asymmetric (3 + 3) cycloaddition of different 2-indolylmethanols

**Fig. 6:**
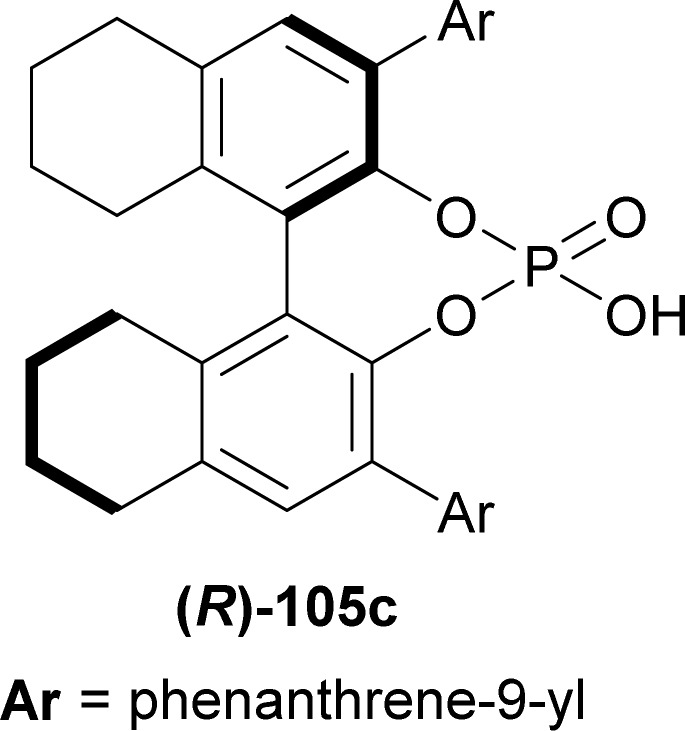
(*R*)-H8-BINOL derived chiral phosphoric acid.

Li *et al.* used (*R*)-H8-BINOL derived catalyst (*R*)-105c (Fig. 6) for the catalytic asymmetric (3 + 3) cycloaddition of (1*H*-indol-2-yl)(2-methoxyphenyl)(phenyl)methanol 117 and (3-methyl-1*H*-indol-2-yl)-(2-methylphenyl)methanol 118.^146^

**Scheme 36:**
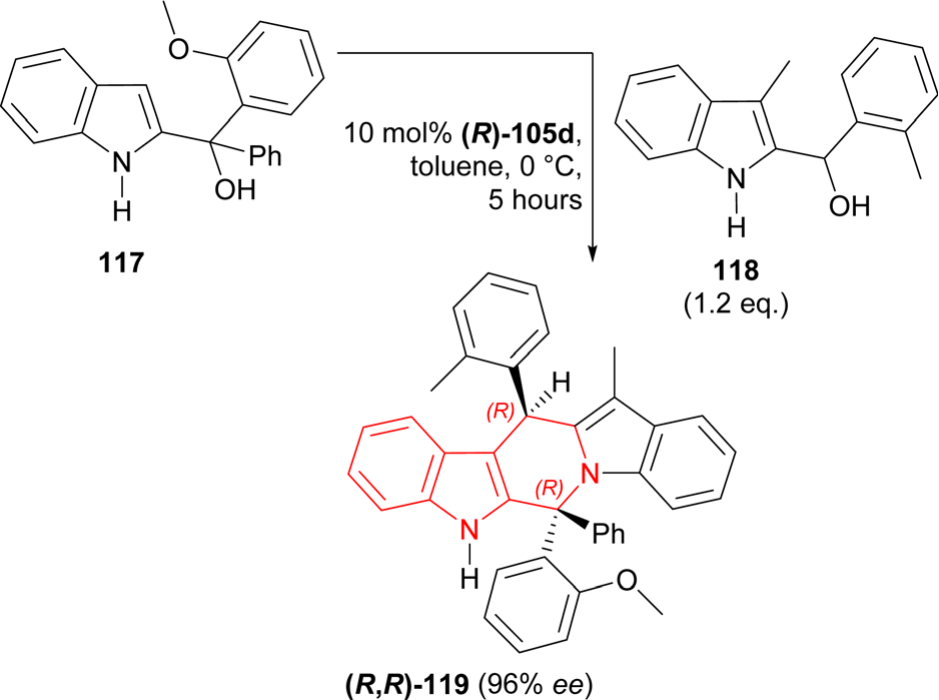
Catalytic asymmetric (3 + 3) cycloaddition of two different 2-indolylmethanols.

117 was stirred with 1.2 eq. 118 and 10 mol% (*R*)-105c for 5 hours in toluene at 0 °C to give catalytic asymmetric (3 + 3) cycloaddition product (6*R*,13*R*)-6-(2-methoxyphenyl)-12-methyl-6-phenyl-13-(2-methylphenyl)-6,13-dihydro-5*H*-pyrido[1,2-*a*:5,4-*b*′]diindole (*R*,*R*)-119 in 57% yield and 96% ee (Scheme 36).

So, (*R*)-configured H8-BINOL derived catalyst gave (*R*,*R*)-configured catalytic asymmetric (3 + 3) cycloaddition product.

### Method 5. Enzymatic catalysis

Biocatalysis have already been employed in a variety of synthesis methods throughout the last few decades.^147,148^ Enzymes are proteins that activate any reaction process by binding to a particular location on the substrate. They are active at mild reaction conditions (pH, temperature, and reaction media *e.g.*, water). These multifunctional catalysts enable several complex chemical processes to be performed in very mild conditions while maintaining excellent activity, selectivity, and specificity.^149,150^ For these reasons, enzymes are essential for the production of chiral building blocks, enantiopure medicines, and pharmaceuticals.^150–154^

#### Example 1. Asymmetric reduction by imine reductase (IRED), freshly prepared or 24 hours old aliquot, from *Amycolatopsis orientalis*

Aleku *et al.* used imine reductase (IRED),^155–157^ freshly purified from *Amycolatopsis orientalis*, *Ao*IRED (UniProt: R4SNK4) to reduce 1-methyl-DHβC 120a and 1-cyclohexyl-DHβC 120b to (1*R*)-1-methyl-THβC (1*R*)-121a and (1*R*)-1-cyclohexyl-THβC (1*R*)-121b respectively.^158^(1*R*)-121a had >99% ee but only 6% conversion while (1*R*)-121b had 71% ee with 66% conversion. But 24 h old aliquot of *Ao*IRED reduced 7-methoxy-1-methyl-4,9-dihydro-3*H*-pyrido[3,4-*b*]indole 120c to (1*S*)-7-methoxy-1-methyl-THβC (1*S*)-121c with the highest ee of 79% (15% conversion).

**Scheme 37:**
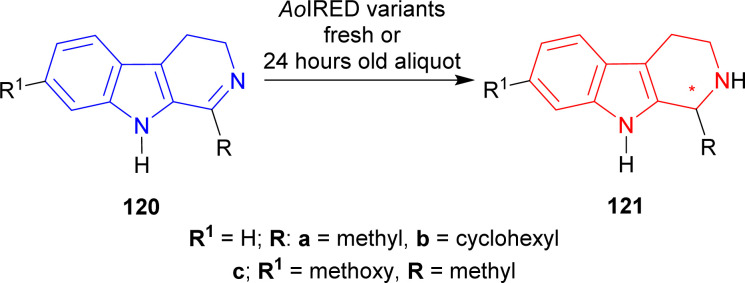
Reduction of 1-substituted-DHβC with fresh or 24 hours old aliquot of *Ao*IRED variants.

Then, six different variants of *Ao*IRED were used to reduce 120c. Only *Ao*IRED N241A produced (1*R*)-7-methoxy-1-methyl-THβC (1*R*)-121c with 96% conversion and 60% ee. *Ao*IRED N171D, *Ao*IRED Y179F, and *Ao*IRED Y179A produced 99% ee of (1*S*)-121c with low conversion of 6, 9, and 10% respectively. *Ao*IRED WT and *Ao*IRED N171A slightly increased % conversion to 15 and 18 but ee decreased to 62 and 71 respectively (Scheme 37).

So, freshly purified *Ao*IRED and *Ao*IRED N241A produced (1*R*)-1-substituted-THβCs; 24 h old aliquot of *Ao*IRED and *Ao*IRED N171D, *Ao*IRED Y179F, *Ao*IRED Y179A, *Ao*IRED WT, *Ao*IRED N171A produced (1*S*)-1-substituted-THβCs predominantly.

#### Example 2. Asymmetric reduction by IREDs of D-type and Y-type

Velikogne *et al.* examined D-type IREDs such as IRED-A–IRED-H and Y-type IREDs such as IRED-I–IRED-N to reduce 1-methyl-4,9-dihydro-3*H*-pyrido[3,4-*b*]indole 120a and 7-methoxy-1-methyl-4,9-dihydro-3*H*-pyrido[3,4-*b*]indole 120c with NADP^+^, *Lactobacillus brevis* alcohol dehydrogenase (*Lb*-ADH), pH 7.5 Tris–HCl buffer or pH 6.0 potassium phosphate buffer, 5% (v/v) IPA, 30 °C, 24 hours.^159^

Among the Y-type IREDs, IRED-J (UniProt: D2PR38, collected from *Kribbella flavida* DSM 17836),^160^ IRED-K (UniProt: D2AWI4, collected from *Streptosporangium roseum* DSM 43201), IRED-L (UniProt: K0F8R0, collected from *Nocardia brasiliensis* ATCC 700358), and IRED-M (UniProt: K0K4C6, collected from *Saccharothrix espanaensis* ATCC 51144) produced (1*S*)-1-methyl-THβC (1*S*)-121a of 91–92% conversion rate (96 to >99% ee) and (1*S*)-7-methoxy-1-methyl-THβC (1*S*)-121c of 95–88% conversion rate (92 to >99% ee). IRED-I (from *Streptomyces* sp. GF3546, UniProt: M4ZS15)^157^ produced >99% ee for both products but (1*S*)-121a had 90% conversion while (1*S*)-121c had only 9% conversion; and IRED-N (from *Bacillus cereus*, UniProt: J7YM26)^161^ showed very little conversion (5 and 2% respectively) for both.

**Scheme 38:**
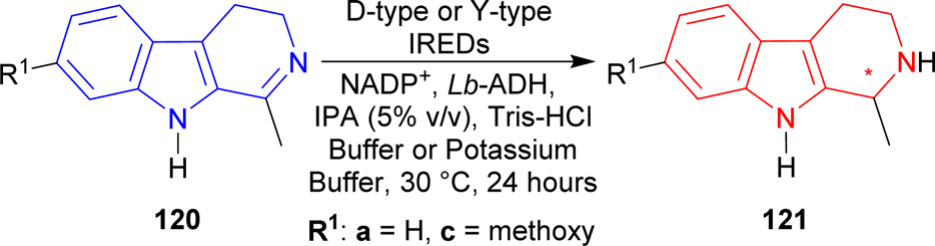
Reduction of 1-substituted-DHβC with D-type IREDs *e.g.*, IRED-A–IRED-H, and Y-type IREDs *e.g.*, IRED-I–IRED-N.

The D-type IREDs did not show any good activity at all for 120c. For 120a, IRED-A (UniProt: M4ZRJ3, collected from *Streptomyces* sp. GF3587)^156^ and IRED-G (UniProt: L8EIW6, collected from *Streptomyces rimosus* ATCC 10970) gave (1*S*)-1-methyl-THβC (1*S*)-121a in 8, 21% conversion and 93, >99% ee respectively; IRED-D (UniProt: V7GV82, collected from *Mesorhizobium* sp. L2C089B000) and IRED-F (UniProt: V6KA13, collected from *Streptomyces niveus* NCIMB 11891) gave (1*R*)-1-methyl-THβC (1*R*)-121a in 27, 13% conversion and 78, >99% ee respectively. IRED-B (UniProt: Q1EQE0, collected from *Streptomyces kanamyceticus*)^162^ did not give any product at all for both reactions while IRED-C (UniProt: W7VJL8, collected from *Micromonospora* sp. M42) did the same as above for 120a and only had 1% conversion for its product. IRED-E (UniProt: J7LAY5, collected from *Nocardiopsis alba*) and IRED-H (UniProt: I8QLV7, collected from *Frankia* sp. QA3) gave only 1% conversion for both of their products (Scheme 38).

So, IRED-A, IRED-G, IRED-I–IRED-N produced (1*S*)-1-substituted-THβCs; and IRED-D, IRED-F produced (1*R*)-1-substituted-THβCs predominantly.

#### Example 3. Stereoselective condensation by strictosidine synthase from *Catharanthus roseus* (*Cr*STR), *Ophiorrhiza pumila* (*Op*STR), *Rauwolfia serpentina* (*Rs*STR) and its V208A variant (*Rv*STR)

**Scheme 39:**
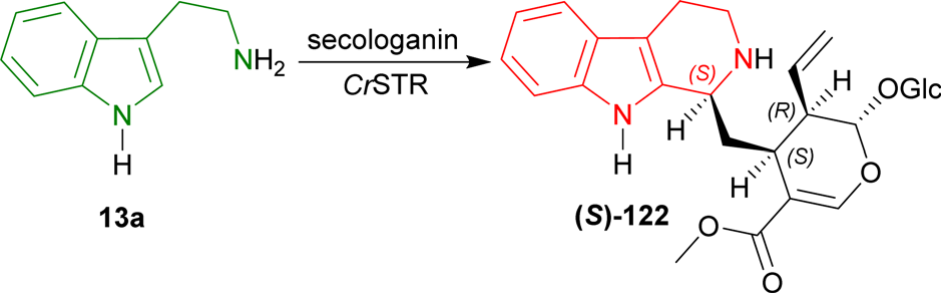
Stereoselective condensation of tryptamine and secologanin with *Cr*STR.

In 1977, Stöckigt and Zenk used strictosidine synthase (STR, EC 4.3.3.2) from *Catharanthus roseus* for stereoselectively condensing tryptamine 13a with secologanin to produce (*S*)-strictosidine (*S*)-122 for the very first time (Scheme 39).^163^

Pressnitz *et al.* tested *Cr*STR, *Op*STR, *Rs*STR, and *Rv*STR for the stereoselective condensation of tryptamine 13a and five small aliphatic aldehydes that gave (1*R*)-1-substituted-THβCs (1*R*)-123a–e as products.^164^

14–38% conversion and 28–43% ee was seen for (1*R*)-123a in case of acetaldehyde as the aliphatic aldehyde. Even, racemic 123a was found for *Cr*STR and *Rs*STR. The ee improved to 61–91% for (1*R*)-123b when butanal was used. Overall conversion also increased to 7–45%. Decreased conversion of 4–8% and ee of 46–82% was seen for (1*R*)-123c when hexanal was used. So, carbon number in the aliphatic aldehyde was not increased further.

After that, steric hindrance in the aliphatic chain of the aldehyde was increased by the use of 3-methylbutanal instead of butanal. Improved 12–77% conversion and 88 to >98% ee was seen for (1*R*)-123d compared to (1*R*)-123b.

Lastly, methyl 4-oxobutanoate was used to asymmetrically condense with 13a. The product (1*R*)-123e showed >98% ee for all the STRs and overall increased conversion of 11–95% was seen compared to (1*R*)-123b.

**Scheme 40:**
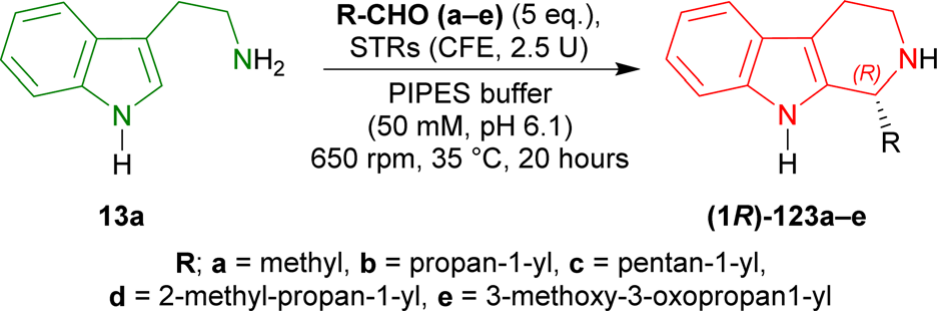
Stereoselective condensation of tryptamine and small aliphatic aldehydes with *CrSTR*, *OpSTR*, *RsSTR*, and *RvSTR*.

*Cr*STR had generally much lower conversion and ee than any other STRs. While *Rv*STR showed highest ee in each product, *Rs*STR had highest conversion for only (1*R*)-123a,c,e. *Rs*STR and *Rv*STR had same or almost similar conversion and ee for (1*R*)-123b,c. *Rs*STR had higher conversion compared to *Op*STR for each product except (1*R*)-123c. *Rv*STR had higher or same ee compared to *Op*STR for each product (Scheme 40).

So, *CrSTR* produced (*S*)-strictosidine with secologanin, while *CrSTR*, *OpSTR*, *RsSTR*, and *RvSTR* produced (1*R*)-1-substituted-THβCs predominantly with small aliphatic aldehydes.

#### Example 4. Asymmetric synthesis of fused-ring THβCs by imine reductases

Yang *et al.* used four IREDs to enantioselectively reduce iminium salts, produced from the Bischler–Napieralski cyclization of hydroxamides, derived from tryptamine and 5-substituted tryptamines.^165^

13a,b,k,l was stirred with 1.1 eq. of oxolan-2-one (γ-butyrolactone) 86a^1^ or oxan-2-one (δ-valerolactone) 86a^2^ and *p*-TsOH in toluene, refluxed for 7 hours to produce hydroxamides 87aa^1,2^,ba^1,2^,ka^1,2^,la^1,2^. Then they were stirred with POCl_3_ in toluene, refluxed for 2 hours to get iminium salts 88aa^1,2^,ba^1,2^,ka^1,2^,la^1,2^ in 58–72% yields by Bischler–Napieralski cyclization.^110,166^ After that, the iminium salts were asymmetrically reduced by IREDs named IR51 (from *Myxococcus fulvus*, Protein Identifier: WP_074958336.1), IR64 (from *Actinocorallia populi*, Protein Identifier: WP_106402132.1), IR86 (from *Paenibacillus lactis*, Protein Identifier: WP_007130043.1),^167^ and IR88 (Metagenome (pIR23)).^168,169^

IR51 and IR88 produced only (*R*)-configured products. IR51 had highest yield of >98% and 99% ee for (*R*)-89aa^1^; other products (*R*)-89aa^2^,ba^1,2^,ka^1,2^,la^1,2^ had 80–98% yields and 95–99% ee. IR88 showed the best result having 99% ee for all products (*R*)-89aa^1,2^,ba^1,2^,ka^1,2^,la^1,2^ with >98% yields except only (*R*)-89ka^1^ (80–98% yield).

On the other hand, IR64 and IR86 produced only (*S*)-configured products. IR64 did not even react with 88aa^1^,ka^1^. Among the other products, (*S*)-89la^1^ had the lowest ee of 29% with 50–80% yield and (*S*)-89aa^2^ had the highest ee of 99% with 50–80% yield. (*S*)-89ba^2^,ka^2^,la^2^ had the lowest yields of 10–50% with 82–95% ee. IR86 had 99% ee for (*S*)-89aa^2^ with 80–98% yield; 98–99% ee for (*S*)-89ba^1,2^,la^1,2^ with 50–98% yields; 76 and 70% ee for (*S*)-89ka^1,2^ with 10–50% and 80–98% yields respectively; 80–98% yield for (*S*)-89aa^1^ with no ee data reported.

**Scheme 41:**
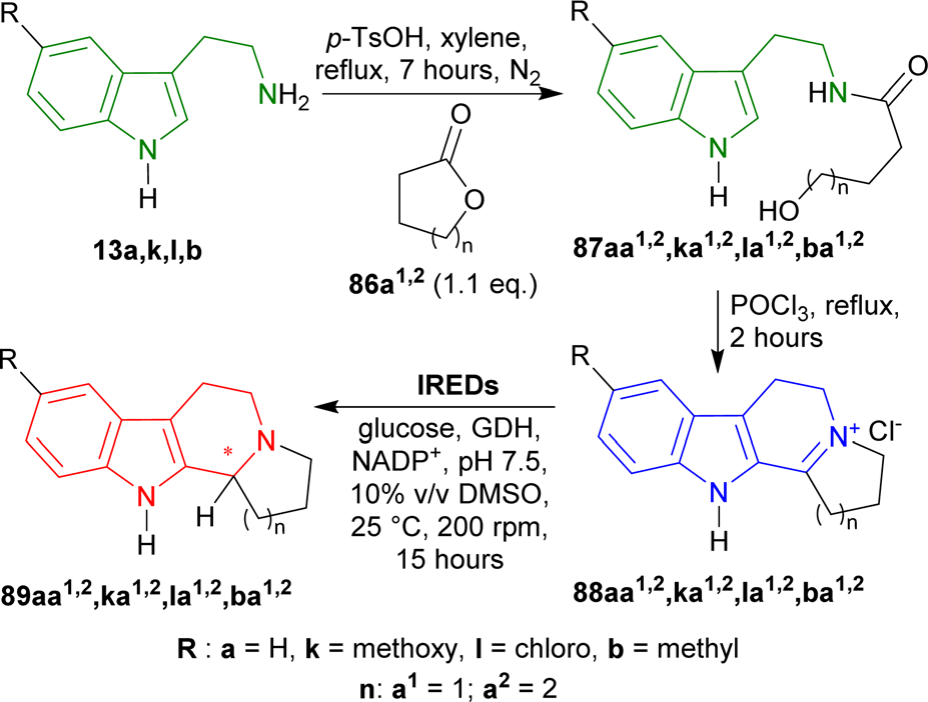
Asymmetrically synthesizing fused-ring THβCs by imine reductases.

Lastly, IR86 and IR88 were used for the preparative scale synthesis of chiral 89aa^1,2^,ba^1,2^,ka^1,2^,la^1,2^. IR86 achieved >98 to >99% ee, 77–95% conversion, and 57–82% yields for (*S*)-89aa^2^,ba^1,2^,la^2^ while, IR88 achieved >99% ee, 79–97% conversion, and 57–74% yields for (*R*)-89aa^1,2^,ba^1,2^,ka^1,2^,la^1,2^ (Scheme 41).

So, IR86 produced (*S*)-configured products and IR88 produced (*R*)-configured products predominantly.

#### Example 5. Asymmetric synthesis of 1-substituted-THβCs by imine reductases

Li *et al.* tested the enzymes IRED-G, IRED-I–IRED-M to reduce 1-*tert*-butyl-4,9-dihydro-3*H*-pyrido[3,4-*b*]indole 18a.^44^

Except IRED-I, others had 97 to >99% conversion of (1*S*)-1-*tert*-butyl-THβC (1*S*)-19a; IRED-G (from *Streptomyces*, Accession No.: WP_003985113.1), IRED-I (from *Streptomyces* sp. GF3546, Accession No.: 4OQY) had 40–41% ee, IRED-J (from *Kribbella flavida*, Accession No.: WP_012921542.1) had 73% ee, IRED-M (from *Saccharothrix espanaensis*, Accession No.: WP_015105194.1) had 97% ee, and IRED-K (from *Streptosporangium roseum*, Accession No.: WP_012890722.1), IRED-L (from *Nocardia brasiliensis*, Accession No.: WP_014988976.1) had the highest 99% ee of (1*S*)-19a.

**Scheme 42:**
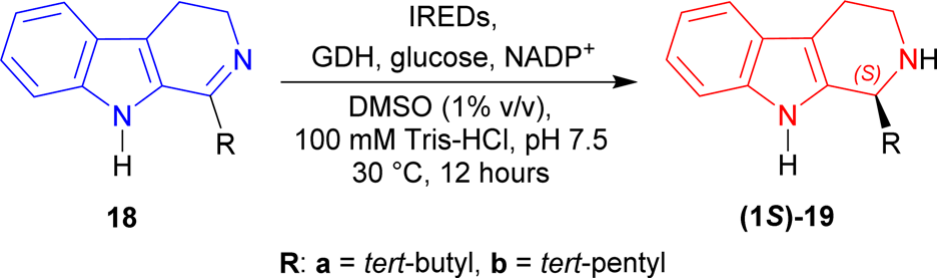
Reduction of 1-substituted-DHβC with IRED-G, IRED-I–IRED-M.

Then, IRED-K–IRED-M were tested on 1-*tert*-pentyl-4,9-dihydro-3*H*-pyrido[3,4-*b*]indole 18b. They produced 79–96% ee but conversion was only 44–70% of (1*S*)-1-*tert*-pentyl-THβC (1*S*)-19b (Scheme 42).

Site-saturation mutagenesis on *At*IRED (from *Amycolatopsis thermoflava*, Accession No.: WP_027931120.1) produced two single mutants named as M118′L and P120′G; one double mutant named as M118′L/P120′G which reduced 18a–i to (1*S*)-19a–i in 98 to >99% ee.

M118′L reduced 1-propyl-4,9-dihydro-3*H*-pyrido[3,4-*b*]indole 18e and 1-cyclopentyl-4,9-dihydro-3*H*-pyrido[3,4-*b*]indole 18g to (1*S*)-1-propyl-THβC (1*S*)-19e and (1*S*)-1-cyclopentyl-THβC (1*S*)-19g respectively in 69% yields, while 1-(2-methylpropyl)-DHβC 18f to 1-(2-methylpropyl)-THβC (1*S*)-19f in 51% yield.

P120′G reduced 18b to (1*S*)-19b in 78% yield, 1-cyclohexyl-DHβC 18h to (1*S*)-1-cyclohexyl-THβC (1*S*)-19h in 64% yield, and 1-phenyl-DHβC 18i to (1*S*)-1-phenyl-THβC (1*S*)-19i in 30% yield.

**Scheme 43:**
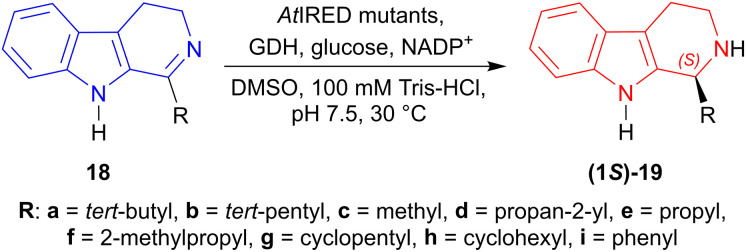
Reduction of 1-substituted-DHβC with *At*IRED mutants.

M118′L/P120′G reduced 18a to (1*S*)-19a in 87% yield, 1-propan-2-yl-DHβC 18d to (1*S*)-propan-2-yl-THβC (1*S*)-19d in 62% yield, and 18c to (1*S*)-1-methyl-THβC (1*S*)-19c in 50% yield (Scheme 43).

So, IRED-G, IRED-I–IRED-M and *At*IRED mutants produced (1*S*)-1-substituted-THβCs predominantly.

## 3. Conclusion

Novel natural and synthetic THβC products continued to be discovered, with ongoing exploration of their biological activity directly related to the C1 chiral center. 1-Substituted-THβCs and their derivatives have diverse biological actions, indicating that they are a promising drug scaffold for treating various diseases. We discussed five synthetic methods with the purpose for creating C1 chiral center. For Pictet–Spengler reaction, the highest yield 99% and >97% ee was found from modified Pictet–Spengler reaction for formal syntheses of (−)-suaveoline, (−)-raumacline, and (−)-*N*^b^-methylraumacline intermediates; for chiral auxiliary, the highest 97% yield and highest 91% ee was reported from asymmetric synthesis of 1-substituted-THβC using pyroglutamic acid derivatives; for ATH with chiral catalysts, the highest afforded 92% yield and highest >98% ee was observed from ATH to synthesize 1-alkyl-1,2,3,4-tetrahydropyrido[3,4-*b*]indole; for asymmetric addition reaction, the highest 91% yield and 96% ee was recorded from synthesis of enantiomer of dihydrocorynantheol; for enzymatic catalysis, the highest conversion of 95% with >98% ee was obtained from stereoselective condensation by STR from *Rauwolfia serpentina*.

The methods that we have discussed here are the most used and widely found pathways for creating C1 chirality which is crucial for prominent biological activities. More efficient and economically feasible pathways should be revised so they could be applied for synthesizing new promising THβCs.

## Data availability

No primary research results, software or code have been included and no new data were generated or analysed as part of this review.

## Author contributions

M. M. A. Asif: writing – original draft, visualization, writing – review & editing. S. R. Lisa: writing – original draft, writing – review & editing. N. Qais: conceptualization, supervision, writing – review & editing.

## Conflicts of interest

There are no conflicts to declare.
